# Molecular docking studies of phytochemicals from *Terminalia chebula* for identification of potential multi-target inhibitors of SARS-CoV-2 proteins

**DOI:** 10.1016/j.jaim.2022.100557

**Published:** 2022-02-16

**Authors:** Arkaniva Sarkar, Rushali Agarwal, Boudhayan Bandyopadhyay

**Affiliations:** a School of Bioscience, Engineering and Technology, VIT Bhopal University, Madhya Pradesh, India; b Department of Biotechnology, School of Life Science and Biotechnology, Adamas University, Kolkata, 700126, India

**Keywords:** SARS-CoV-2, Phytochemical, Molecular docking, Multi-target drug, ACE, Atomic Contact Energy (kcal/mol), ADME/T, Absorption, Distribution, Metabolism, Excretion, and Toxicity, NSP, Non-structural Protein, PDB, Protein Data Bank, *T. chebula*, *Terminalia chebula*

## Abstract

**Background:**

The COVID-19 caused by severe acute respiratory syndrome coronavirus 2 (SARS-CoV-2) has emerged as a global pandemic claiming more than 6 million lives worldwide as of 16 March 2022. Till date, no medicine has been developed which is proved to have 100% efficiency in combating against this deadly disease. We focussed on ayurvedic medicines to identify drug-like candidates for treatment and management of COVID-19. Among all ayurvedic medicines, we were interested in *Terminalia chebula* (T. chebula), as it is known to have antibacterial, antifungal, antiviral, antioxidant and anti-inflammatory properties.

**Objectives:**

In this study, we evaluated potential inhibitory effects of phytochemicals from *T. chebula* against eight structural and functional proteins of SARS-CoV-2.

**Material and methods:**

We performed blind molecular docking studies using fifteen phytochemicals from *T. chebula* against the proteins of SARS-CoV-2. The three-dimensional proteins structures were analysed and potential drug-binding sites were identified. The drug-likeness properties of the ligands were assessed as well.

**Results:**

Analysing the docking results by comparing Atomic Contact Energy (ACE) and intermolecular interactions along with assessment of ADME/T properties identified 1,3,6-Trigalloyl glucose (−332.14 ± 55.74 kcal/mol), Beta-Sitosterol (−324.75 ± 36.98 kcal/mol) and Daucosterol (−335.67 ± 104.79 kcal/mol) as most promising candidates which exhibit significantly high inhibition efficiency against all eight protein targets.

**Conclusions:**

We believe that our study has the potential to help the scientific communities to develop multi-target drugs from *T. chebula* to combat against the deadly pathogen of COVID-19, with the support of extensive wet lab analysis.

## 1 Introduction

The global outbreak of pandemic COVID-19 demands the design and development of effective drugs and vaccines as it has already caused more than 6 million deaths worldwide within a year in spite of all types of efforts from scientific and medical communities [1]. Although some of the vaccines are under phase 3 trials and also the vaccination has just started across the world [2], no specific anti-viral drug are developed till date for the treatment and prevention of COVID-19.

In our study, our focus is on the Indian traditional herbal medicine known as “*Ayurvedic* medicine” as it is completely natural, derived from the plants. In ancient *Vedic* era, the *Ayurvedic* medicine was regularly used for the treatment of fever, flu, cough and cold [3]. Among all *Ayurvedic* medicines, we mainly focussed on *Terminalia chebula* as it is known to possess high therapeutic benefits in the treatment of wide range of diseases. It was also regarded as “*King of Medicines”* because of its efficacy in wide range of traditional remedies and curing several diseases [4]. *T. chebula* is mainly found in south Asiatic countries and belongs to *Terminalia* genus which has 250 species distributed across the tropical region of the world [5]. It is reported that some compounds extracted from *T. chebula* exhibits anti-bacterial and anti-viral activity [6,7]. In a recent study, Upadhyay et al. performed screening of 51 medicinal plants against SARS-CoV-2 targeting its main protease to identify the potential therapeutic medicinal herb and found that Tea (*C. sinensis*) and *Haritaki* (*T. chebula*) exhibits significant inhibitory activities against 3CL^pro^ protease of SARS-CoV-2 [8]. This finding led us to perform the *in silico* screening of phytochemicals from *T. chebula* to identify the potential drug for the treatment and prevention of COVID-19.

In *T. chebula,* a wide range of natural products are present including several flavonoids, flavins, terpenoids, tannins, steroids, phenols [5]. In the present work, we chose fifteen phytochemical molecules from flavonoids, flavins, terpenoids, steroids families (Supplementary Table 1) for our screening purpose, and performed blind docking between the selected phytochemicals and the reported crystal structures of the target proteins of SARS-COV-2 having important functions. The target proteins include nucleocapsid protein N-terminal RNA binding domain [9], NSP15 Endoribonuclease [10], Nsp9 RNA binding protein [11], Papain-like protease [12], Nonstructural protein 10 (NSP10) [13], NSP13 helicase [14], main protease [15] and RNA-dependent RNA polymerase (RdRp) [16]. We analysed for potential inhibitory activity through docking study for all fifteen phytochemicals and found that 1,3,6-Trigalloyl glucose, Beta-Sitosterol and Daucosterol possess most promising inhibitory effect against all eight SARS-CoV-2 proteins. Arjunetin, Arjungenin, Arjunic acid and Arjunolic acid were also found to be effective against at least six proteins. These results suggeste that these phytochemicals can be used as potential multi-target inhibitors against various proteins of SARS-CoV-2, however clinical trials should be conducted to validate these results.

## 2 Materials and methods

### 2.1 Ligand and receptor

The 3D structure of the proteins - nucleocapsid protein N-terminal RNA binding domain (PDB: 6M3M), RNA-dependent RNA polymerase (PDB: 6M71), NSP15 Endoribonuclease (PDB: 6VWW), Nsp9 RNA binding protein (PDB: 6W4B), Papain-like protease (PDB: 6WUU), Nonstructural protein 10 (PDB: 6ZCT), helicase (PDB: 6ZSL) and main protease (PDB: 7COM) of SARS-CoV-2 were retrieved from the Protein Data Bank (https://www.rcsb.org/). The PDB files were cleaned by removing any water molecules and hetero atoms if present.

A library of fifteen phytochemicals which can be extracted from *T. chebula* was created. The ligands 1,3,6-Tri-O-galloyl-beta-d-glucose (PubChem: 452707), Arjunetin (PubChem: 21152828), Arjungenin (PubChem: 12444386), Arjunic acid (PubChem: 15385516), Arjunolic acid (PubChem: 73641), Ascorbic acid (PubChem: 5785), Beta-sitosterol (PubChem: 222284), Chebulic acid (PubChem: 25255065), Daucosterol (PubChem: 5742590), Ellagic acid (PubChem: 5281855), Isoquercitrin (PubChem: 5280804), Isorhamnetin (PubChem: 5281654), Luteolin (PubChem: 5280445), Quercetin (PubChem: 5280343), Rutin (PubChem: 5280805) and Terminolic acid (PubChem: 12314613) were obtained from NCBI PubChem database (https://pubchem.ncbi.nlm.nih.gov/) and were categorised based on the class. The ligands were converted into PDB file format using openbabel software (http://openbabel.org).

### 2.2 Validation of protein quality

The quality of the three-dimensional structures of the proteins were validated using Prosa (https://prosa.services.came.sbg.ac.at/prosa.php) and Procheck (https://servicesn.mbi.ucla.edu/PROCHECK/) server. The Z-score and percentage of residues in favoured regions and in outliers were noted for each protein.

### 2.3 Binding pocket prediction

Potential drug binding pockets, volume and surface to volume ratio of the pockets, drugScore and details of residues present in each pocket are identified using DOGSiteScorer server (https://proteins.plus/). The geometric and physicochemical properties of the binding pockets are analyzed and the druggability is estimated with aid of a support vector machine (SVM). Top three binding pockets are selected based on their drugscore.

### 2.4 Blind molecular docking

Molecular docking was done with the set of 15 ligands to find possible interactions. Patch dock web server (https://bioinfo3d.cs.tau.ac.il/PatchDock/), which works on the basis of shape complementarity approach of molecular docking, was used to perform the docking runs. We opted for blind approach throughout all docking interactions in which there will not be any flexibility in the target proteins and the binding pocket will not be defined, in order to ensure that all the interactions are completely unbiased. The PDB files of the proteins and the ligands were uploaded in place of receptor molecule and ligand molecule respectively. The clustering RMSD was set to 4.0 and the complex type was given as protein-small ligand. Top 100 docked complexes for each protein along with their score, atomic contact energy (ACE) and transformation data were saved. Discovery studio visualizer was used to analyze presence of possible protein-ligand interactions.

### 2.5 ADME/T analysis

The pharmacokinetic properties of major small molecules were predicted with the help of SwissADME server (http://www.swissadme.ch/) and pharmacodynamic properties were predicted using admetSAR server (http://lmmd.ecust.edu.cn/admetsar1/predict/). Pharmacokinetic properties were evaluated using Lipinski's rule of five. Molecules which obeys Lipinski's rule can be considered as ideal drug candidates. Parameters defining absorption, distribution, metabolism, excretion, toxicity, solubility (LogS), human intestinal absorption (HIA), CaCO-2 permeability, P-glycoprotein substrate inhibition, cytochrome substrate/inhibitor, AMES toxicity and acute rat toxicity (LD50) were checked in pharmacodynamic study [17].

## 3 Results

### 3.1 Construction of library of phytochemicals and target proteins

A library of fifteen phytochemicals of *T. chebula* was constructed using literature survey [5,18] (Supplementary Table 1). Their 3D structures were retrieved from NCBI PubChem database and categorized based on their functional groups. ADME/T analysis was performed to predict both pharmacokinetic (Supplementary Table 5.1) and pharmacodynamic properties (Supplementary Table 5.2). The eight proteins of SARS-CoV-2 were selected based on their biological functions. The crystal structures of these proteins were retrieved from Protein Data Bank (Supplementary Table 2) and the structural integrity were checked for each PDB file (Supplementary Table 3). The Z-scores and the percentage of residues in favoured region for each protein suggest that they are structurally good for further *in silico* analysis. The binding pocket for each protein structures were predicted with the help of DOGSiteScorer server and top three major binding pockets were selected for analysis and comparison of the interacting residues in the docking sites (Supplementary Table 4).

Each protein was blindly docked against the library of fifteen ligands and top 100 docked complexes for each protein were generated along with docking score and atomic contact energy (ACE) data. The best docking pose was selected based on docking score, favourable ACE and intermolecular interaction. The details of molecular interactions between each protein target and the ligands are discussed below.

### 3.2 N-terminal RNA binding domain of nucleocapsid protein as drug target

**Fig. 1:**
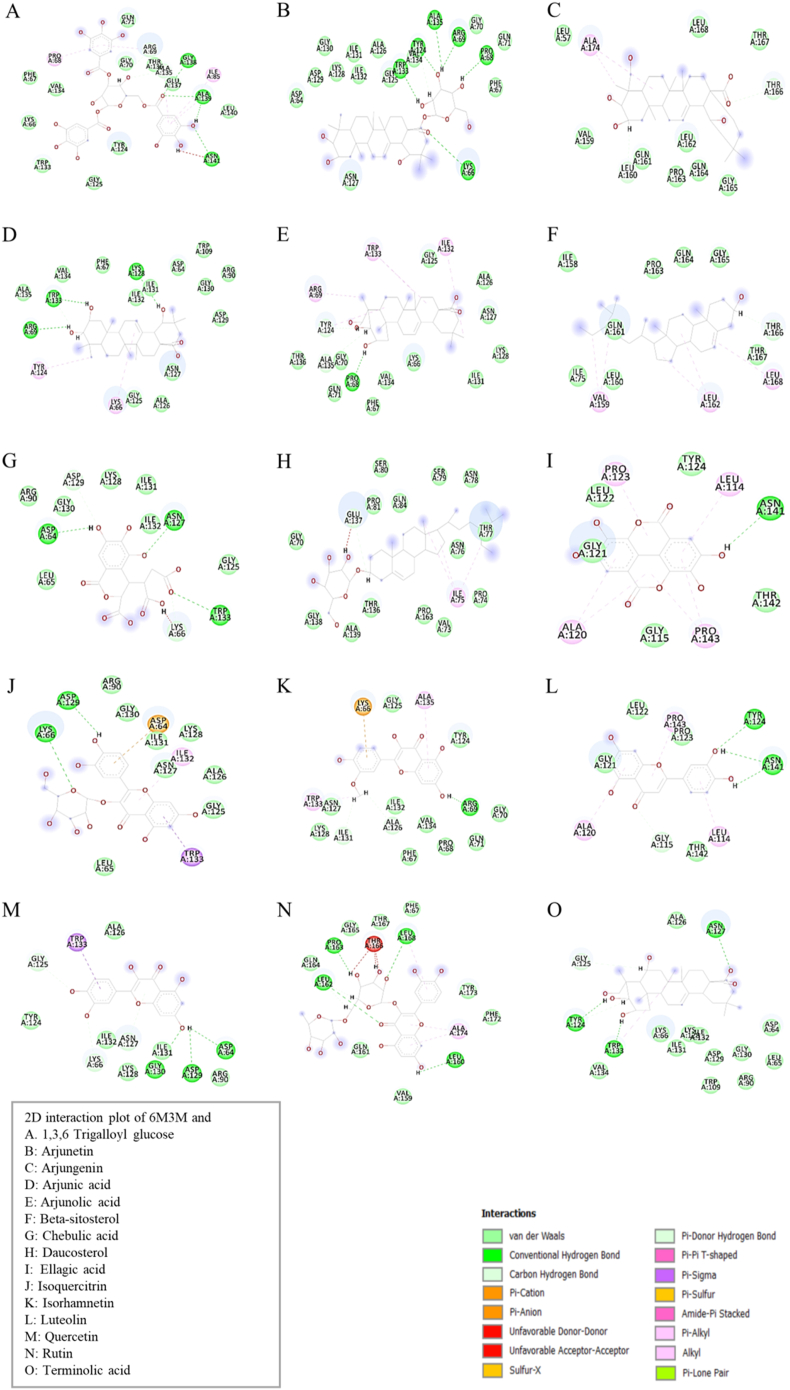
2D interaction plot of interaction site of docking between 6M3M (N-terminal RNA binding domain of Nucleocapsid protein) and the following phytochemicals: A. 1,3,6 Trigalloyl glucose, B. Arjunetin, C. Arjungenin, D. Arjunic acid, E. Arjunolic acid, F. Beta-sitosterol, G. Chebulic acid, H. Daucosterol, I. Ellagic acid, J. Isoquercitrin, K. Isorhamnetin, L. Luteolin, M. Quercetin, N. Rutin and O. Terminolic acid. All the interactions represented by different colour schemes are shown at the right hand side bottom corner in the figure.

The nucleocapsid protein plays a crucial role in transcription and translation of viral RNA. It helps formation of ribonucleoproteins during viral assembly, assists in viral RNA synthesis and affects host cell responses [9]. The N-terminal RNA binding domain of this protein is structurally distinct compared to that of other RNA binding proteins. Residues of N-terminal RNA binding domain of Nucleocapsid protein involved in RNA-binding are Leu56, Gly60, Lys61, Lys65, Phe66, Ala90, Arg93, Ile94, Arg95, Lys102, Asp103, Leu104, Thr165, Thr166, Gly175 and Arg177 [19]. Our docking study using the crystal structure of this N-terminal RNA binding domain (PDB ID: 6M3M) indicated that Beta-Sitosterol and 1,3,6-Trigalloyl glucose shows the most promising inhibition property as evident from their ACE data i.e. −326.98 kcal/mol and −325.98 kcal/mol respectively (Supplementary Table 6). Beta-Sitosterol mainly interacts with the alkyl groups of hydrophobic residues (Leu168, Leu162, Val159), whereas 1,3,6-Trigalloyl glucose through both hydrogen bonding (H-bonding) (Gly138, Ala139, Asn141) and alkyl groups of hydrophobic residues (Pro68, Arg69, Ala135, Ile85, Ala139) of the protein as shown in the 2D interaction plot (Fig. 1F and A, Supplementary Table 6). Pro68, Arg69, Ala135 are part of the predicted binding pocket in N-terminal RNA binding domain. Although Daucosterol also exhibit energetically favourable interaction with 6M3M, no favourable interaction such as H-bonding, alkyl hydrophobic interaction between the ligand and the protein was observed in the docking pose. Apart from 1,3,6-Trigalloyl glucose and beta-Sitosterol; Arjunolic acid, Ellagic acid, Luteolin and Rutin show moderate inhibitory properties in terms of ACE (<-200 kcal/mol) and favourable H-bonding and alkyl interactions with 6M3M. All these molecules bind near the RNA-binding residues which indicate that these molecules may inhibit the RNA binding activity of this protein through competitive manner.

### 3.3 NSP15 endoribonuclease as drug target

**Fig. 2:**
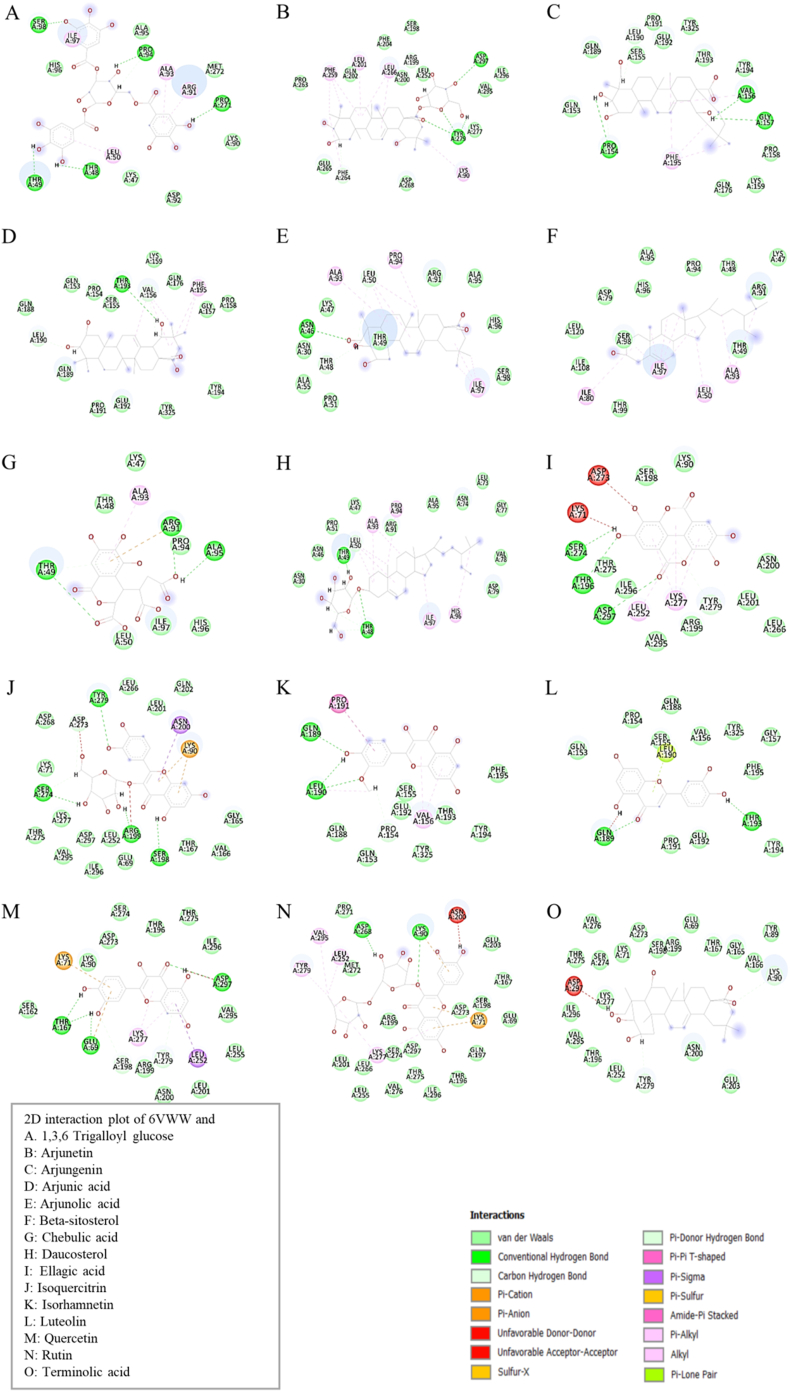
2D interaction plot of interaction site of docking between 6VWW (NSP15 Endoribonuclease) and the following phytochemicals: A. 1,3,6 Trigalloyl glucose, B. Arjunetin, C. Arjungenin, D. Arjunic acid, E. Arjunolic acid, F. Beta-sitosterol, G. Chebulic acid, H. Daucosterol, I. Ellagic acid, J. Isoquercitrin, K. Isorhamnetin, L. Luteolin, M. Quercetin, N. Rutin and O. Terminolic acid. All the interactions represented by different colour schemes are shown at the right hand side bottom corner in the figure.

NSP15 is responsible for the protein interference with the innate immune response of the host. It is also suggested that it breaks down the viral RNA to hide it from host immune responses [10]. The *in silico* screening of fifteen phytochemicals was performed against the crystal structure of NSP15 Endoribonuclease (PDB ID: 6VWW). The docking results indicated that Beta-Sitosterol, 1,3,6-Trigalloyl glucose, Daucosterol and Arjunolic acid shows significantly higher inhibition property among 15 ligands as evident from their ACE data i.e. −343.98, −339.28, −332.89 and −327.48 kcal/mol respectively (Supplementary Table 7). Beta-Sitosterol (Ile80, Ile97, Leu50, Ala93) and Arjunolic acid (Ala93, Leu50, Pro94, Ile97) mainly interacts with the alkyl groups of hydrophobic residues of 6VWW as shown in the 2D interaction plot (Fig. 2F and E, Supplementary Table 7), whereas 1,3,6-Trigalloyl glucose and Daucosterol interacts through H-bonding and alkyl hydrophobic interactions (Fig. 2A and H, Supplementary Table 7). In case of 1,3,6-Trigalloyl glucose, Ser98, Pro94, Pro271, Thr48, Thr49 residues of the protein are involved in H-bonding and Ile97, Ala93, Arg91, Leu50 residues are involved in hydrophobic interaction (Fig. 2A, Supplementary Table 7). In case of Daucosterol, it is mainly hydrophobic interaction which takes place with His96, Ile97, Pro94, Ala93, Leu50 residues of the protein (Fig. 2H, Supplementary Table 7). Arjunetin (−294.42 kcal/mol), Arjungenin (−297.74 kcal/mol), Arjunic acid (−281.34 kcal/mol), Isorhamnetin (−213.7 kcal/mol) and Luteolin (−244.27 kcal/mol) also exhibit moderate inhibition efficiency apart from those above-mentioned top four inhibitors (Supplementary Table 7). It is observed that all these molecules are found to bind to the middle domain of NSP15, which is responsible for hexamer formation and trimer stability [20,21].

### 3.4 Nsp9 RNA binding protein as drug target

**Fig. 3:**
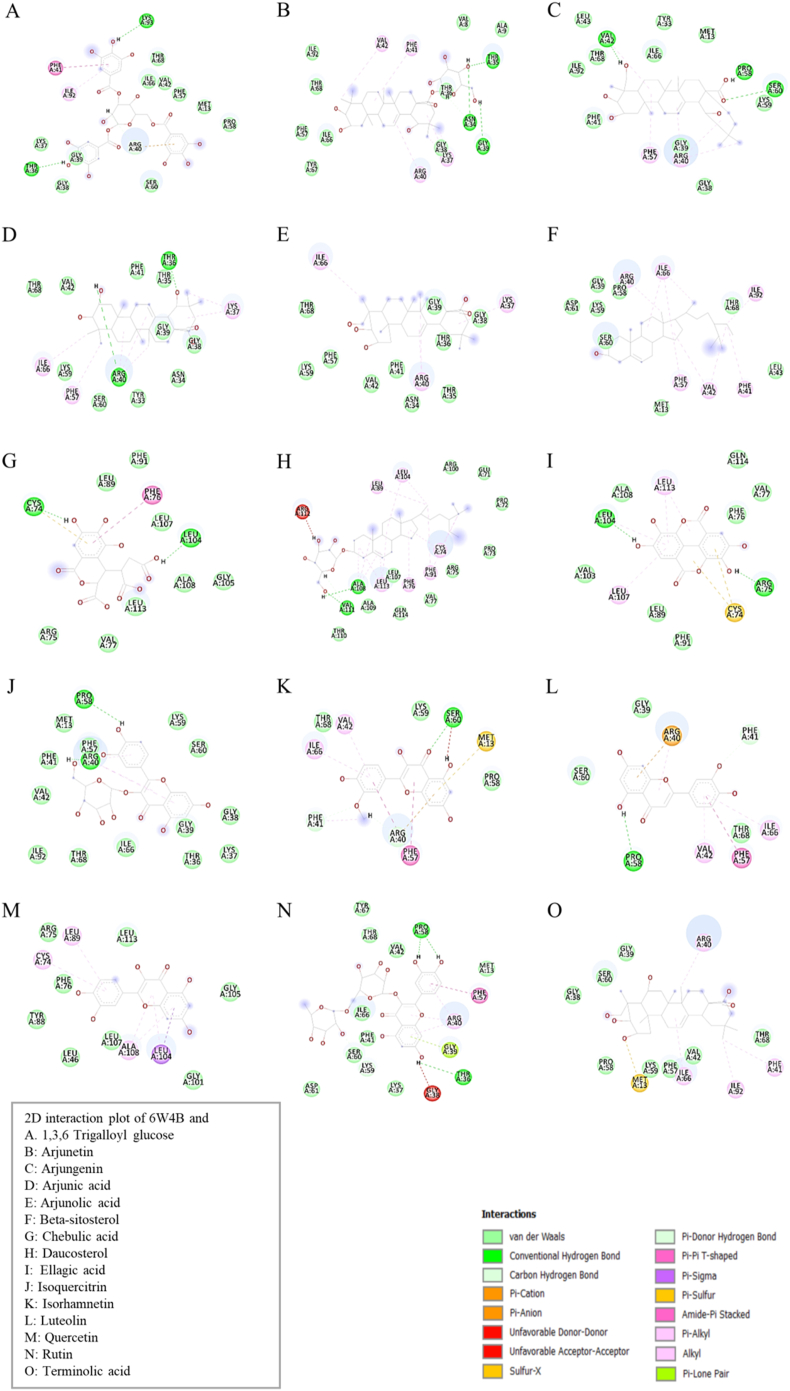
2D interaction plot of interaction site of docking between 6W4B (Non-structural protein 9: Nsp9) and the following phytochemicals: A. 1,3,6 Trigalloyl glucose, B. Arjunetin, C. Arjungenin, D. Arjunic acid, E. Arjunolic acid, F. Beta-sitosterol, G. Chebulic acid, H. Daucosterol, I. Ellagic acid, J. Isoquercitrin, K. Isorhamnetin, L. Luteolin, M. Quercetin, N. Rutin and O. Terminolic acid. All the interactions represented by different colour schemes are shown at the right hand side bottom corner in the figure.

NSP9 is involved in viral RNA synthesis and mediates viral replication [11]. Although the RNA binding mechanism is not clearly known, it is important for the virulence of SARS-CoV-2. The crystal structure of NSP9 (PDB ID: 6W4B) was used for our docking study. The docking results indicate that Arjunetin (−464.74 kcal/mol) and Daucosterol (−409.83 kcal/mol) are the top two inhibitors as evident from the highly favourable ACE data i.e −464.74 and −409.83 kcal/mol respectively. These two ligands interact mainly with the alkyl groups of hydrophobic residues (situated in the predicted binding pocket) of Nsp9 along with H-bonding as shown in (Fig. 3B and H) and Supplementary Table 8. In this case, on an average, all the ligands except Luteolin (−181.15 kcal/mol) interact with Nsp9 with favourable ACE (<- 200 kcal/mol). In most of the cases, the interaction happened primarily through alkyl hydrophobic interaction along with less number of H-bonding as shown in 2D interaction plot (Fig. 3) and Supplementary Table 8. In this case, all these molecules bind to a region 11.92 ± 3.3 Å apart from G-x-x-x-G motif (Gly101, Met102, Val103, Leu104, Gly105) which is responsible for its dimerization [22]. This type of interaction is indicative of possible non-competitive inhibition against the NSP9 activity.

### 3.5 Papain-like protease as drug target

**Fig. 4:**
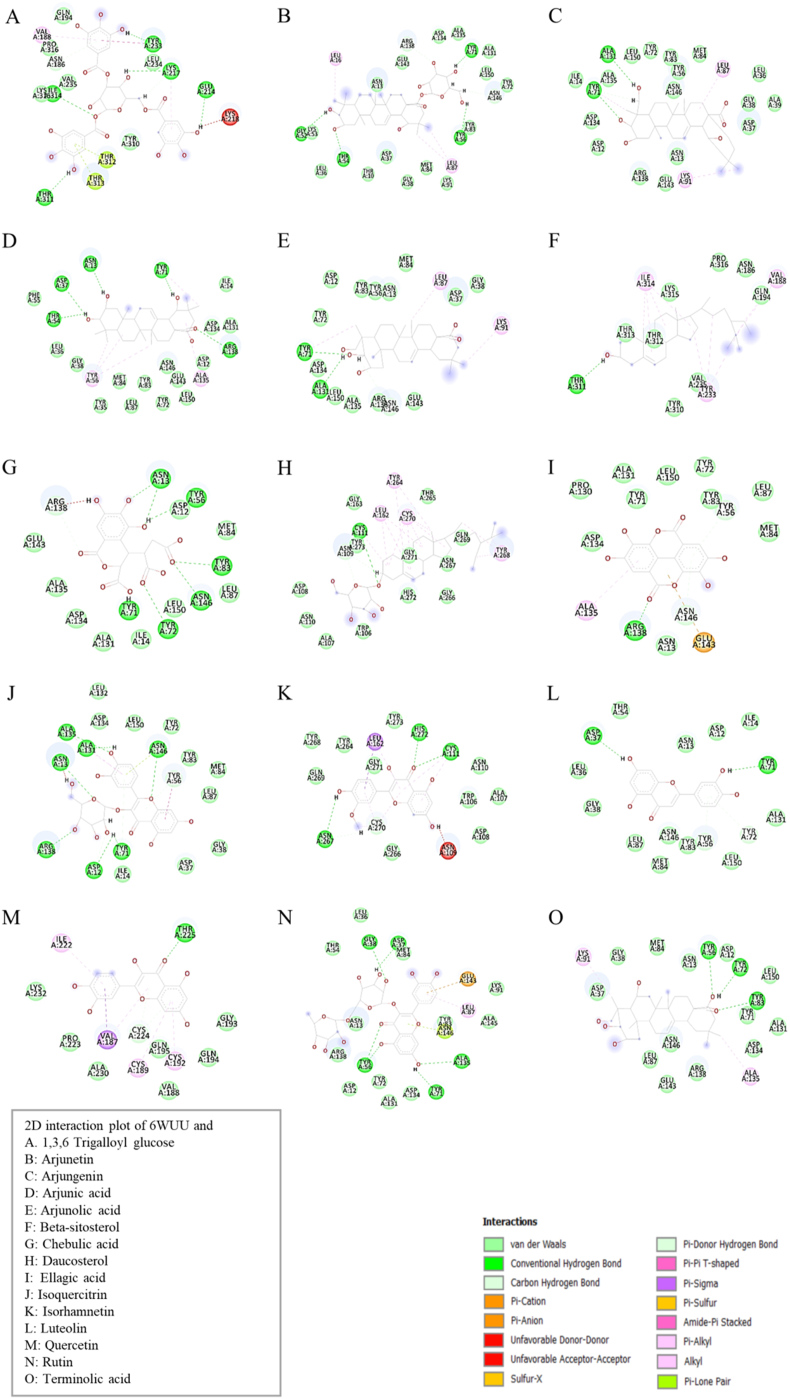
2D interaction plot of interaction site of docking between 6WUU (Papain-like protease) and the following phytochemicals: A. 1,3,6 Trigalloyl glucose, B. Arjunetin, C. Arjungenin, D. Arjunic acid, E. Arjunolic acid, F. Beta-sitosterol, G. Chebulic acid, H. Daucosterol, I. Ellagic acid, J. Isoquercitrin, K. Isorhamnetin, L. Luteolin, M. Quercetin, N. Rutin and O. Terminolic acid. All the interactions represented by different colour schemes are shown at the right hand side bottom corner in the figure.

The papain-like protease is responsible for processing viral polyproteins and viral replication. It is also known to regulate SARS-CoV-2 viral spread and innate immunity [12]. The crystal structure of Papain-like protease (PDB ID: 6WUU) was used for docking against fifteen phytochemicals (Supplementary Table 9). The results indicate that Daucosterol is the best candidate against Papain-like protease as evident from its highly energetically favourable interaction (ACE: −520.75 kcal/mol). It mainly interacts mainly with the hydrophobic residues (Cys111, Leu162, Cys270, Tyr264, Tyr268), residing in the active site of 6WUU (catalytic triad composed of Cys112–His273–Asp287) [23], as visible in the 2D interaction plot (Fig. 4H) and Supplementary Table 9. Beta-Sitosterol also exhibits considerably favourable binding through hydrophobic interactions with Ile314, Val188, Tyr233 residues of the protein and low ACE (−363.05 kcal/mol) (Fig. 4F and Supplementary Table 9), although the interaction is far apart from the catalytic triad suggesting possible non-competitive inhibition against the protein function. Apart from these two phytochemicals, 1,3,6-Trigalloyl glucose show moderately high interaction with 6WUU (ACE: −260.13 kcal/mol) through high number of H-bonding (Glu214, Lys217, Tyr233, Ile314, Thr311) and hydrophobic interaction (Thr313, Thr312, Asn186, Val188, Tyr233, Lys217) (Fig. 4A and Supplementary Table 9). Similar to Beta-Sitosterol, in this case the inhibition seems to take place in non-competitive manner. Isorhamnetin (−255.64 kcal/mol) and Quercetin (−256.38 kcal/mol) exhibit moderate inhibition property mainly through hydrophobic interaction (Fig. 4K and M and Supplementary Table 9) with the protein. Isorhamnetin competes at catalytic site of 6WUU, whereas Quercetin interacts in non-competitive manner.

### 3.6 Non-structural protein 10 (nsp10) as drug target

**Fig. 5:**
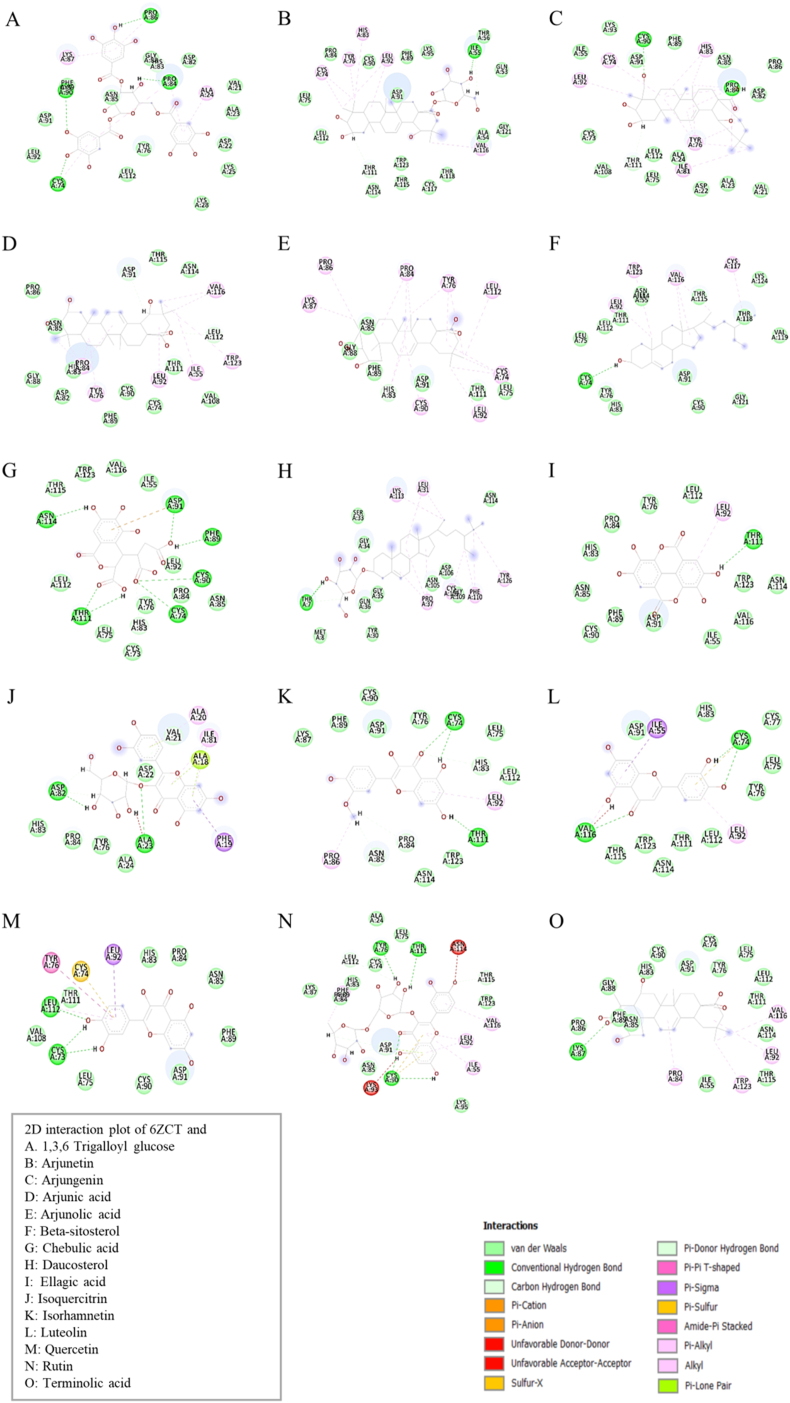
2D interaction plot of interaction site of docking between 6ZCT (Non-structural protein 10: nsp10) and the following phytochemicals: A. 1,3,6 Trigalloyl glucose, B. Arjunetin, C. Arjungenin, D. Arjunic acid, E. Arjunolic acid, F. Beta-sitosterol, G. Chebulic acid, H. Daucosterol, I. Ellagic acid, J. Isoquercitrin, K. Isorhamnetin, L. Luteolin, M. Quercetin, N. Rutin and O. Terminolic acid. All the interactions represented by different colour schemes are shown at the right hand side bottom corner in the figure.

NSP10 is a small stimulatory and scaffolding protein which stimulates exoribonuclease activity and plays a key role in RNA methylation machinery [13]. NSP10 consists of two zinc binding sites, one with residues Cys74, Cys77, Cys90, and His83, which stabilizes the α2 and α3 helices, and the other with four cysteine residues, Cys117, Cys120, Cys128, Cys130, which stabilizes the C-terminal of the NSP10 protein [24]. The docking study was performed using the crystal structure of NSP10 (PDB ID: 6ZCT) against the library of phytochemicals. In this case, mostly all the ligands interacted with highly favourable ACE (<-200 kcal/mol) except Ellagic acid and Isorhamnetin. Based on the ACE data, it was observed that Arjungenin (−467.1 kcal/mol), Arjunolic acid (−457.06 kcal/mol) and Terminolic acid (−456.93 kcalmol) are the top three inhibitors (Supplementary Table 10). All these three phytochemicals mainly interact with hydrophobic residues of NSP10 as visible from the 2D interaction plot (Fig. 5C, E and 5O) and Supplementary Table 10. The participating hydrophobic residues lie within the predicted binding pocket in each case. 1,3,6-Trigalloyl glucose (−397.95 kcalmol), Rutin (−359.1 kcalmol), Isoquercitrin (−344.28 kcalmol) and Arjunic acid (−334.02 kcalmol) also exhibit promising inhibitory feature based on their ACE data (Supplementary Table 10) and favourable interaction with NSP10 (Supplementary Table 10). 1,3,6-Trigalloyl glucose interacts through both H-Bonding with Pro86, Pro84, Cys90, Cys74 and hydrophobic interaction with residues Lys87, Ala24, Pro84, Cys74, Pro86 (Fig. 5A; Supplementary Table 10). In case of Isoquercitrin, it is mainly hydrophobic interaction which takes place with Phe19, Ala18, Ile81, Ala20 residues (Fig. 5J; Supplementary Table 10). In case of Arjunic acid, only hydrophobic interaction takes place with residues Pro84, Tyr76, Leu92, Ile55, Trp123, Val116 (Fig. 5D; Supplementary Table 10). All these molecules are found to bind to the zinc binding site of NSP10 protein suggesting strong competitive inhibition against the protein activity.

### 3.7 Helicase protein (NSP13) as drug target

**Fig. 6:**
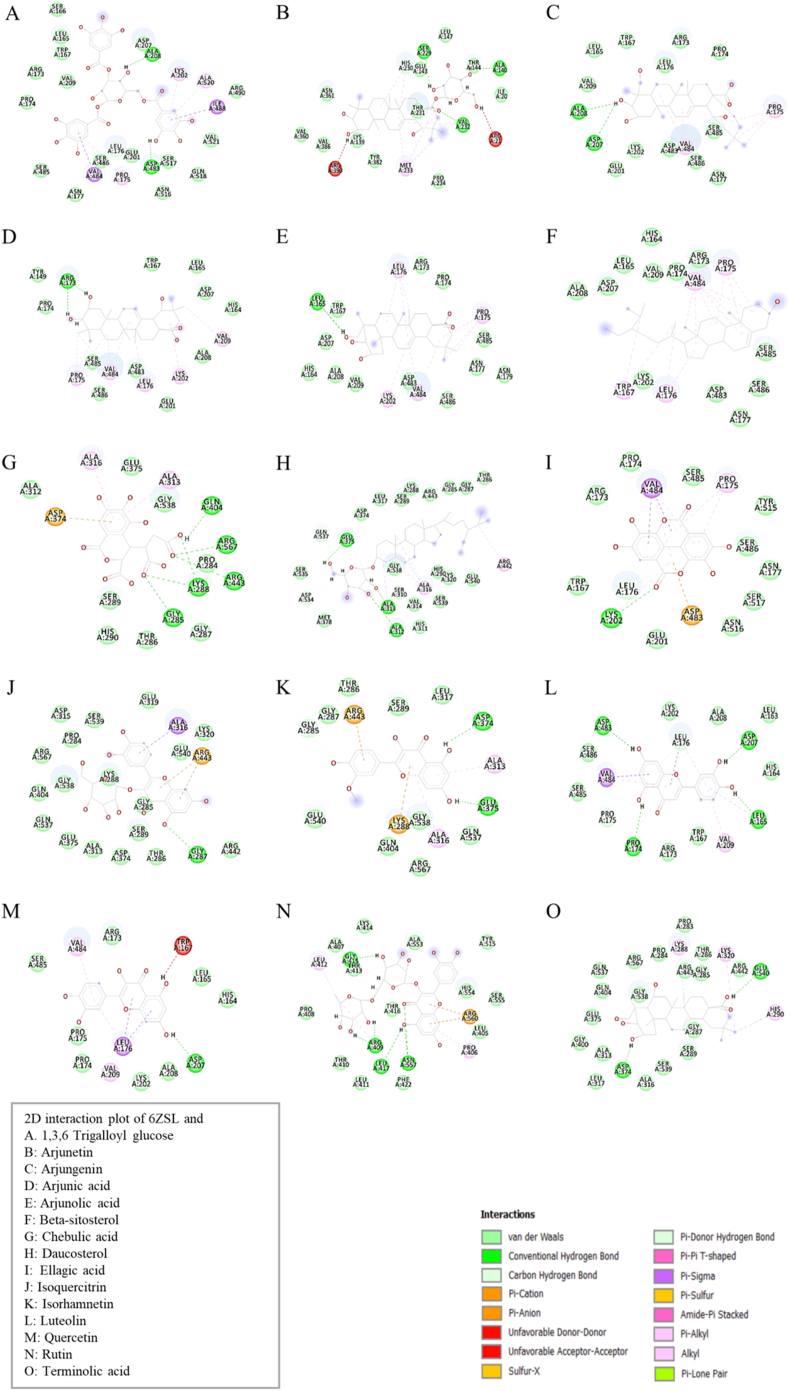
2D interaction plot of interaction site of docking between 6ZSL (Helicase) and the following phytochemicals: A. 1,3,6 Trigalloyl glucose, B. Arjunetin, C. Arjungenin, D. Arjunic acid, E. Arjunolic acid, F. Beta-sitosterol, G. Chebulic acid, H. Daucosterol, I. Ellagic acid, J. Isoquercitrin, K. Isorhamnetin, L. Luteolin, M. Quercetin, N. Rutin and O. Terminolic acid. All the interactions represented by different colour schemes are shown at the right hand side bottom corner in the figure.

NSP13 Helicase is important for viral replication and proliferation [14]. The crystal structure of NSP13 Helicase (PDB ID: 6ZSL) was used for docking study. Arjunetin was identified as best inhibitor based on lowest ACE i.e. −393.3 kcal/mol. It interacts through both H-bonding with Ser229, Ala140, Val232 and hydrophobic interaction with residues His230, Met233, Val232 (Fig. 6B; Supplementary Table 11). Arjunolic acid (−379.29 kcal/mol), 1,3,6-Trigalloyl glucose (−339.91 kcal/mol), Beta-Sitosterol (−361.93 kcal/mol), Arjunic acid (−316.8 kcal/mol) and Arjungenin (−314.33 kcal/mol) also exhibit promising inhibitory effect against NSP13 Helicase as evident from Supplementary Table 11, and have favourable hydrophobic interactions (Fig. 6E, A, 6F, 6D and 6C respectively). All those molecules are found to bind to a region 16.39 ± 8.1 Å apart from the active site of NSP13 [24] indicating non-competitive type of inhibition.

### 3.8 Main protease (7COM) as drug target

**Fig. 7:**
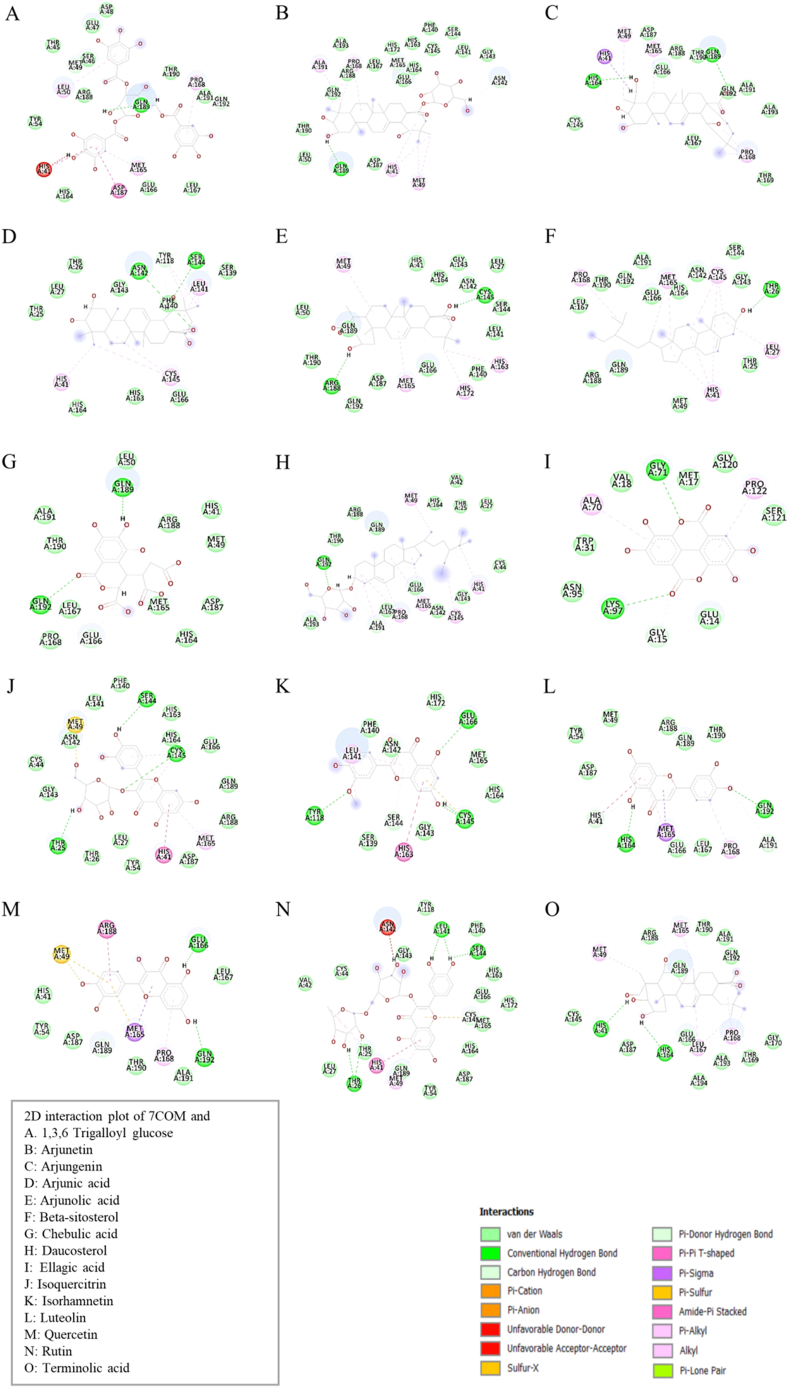
2D interaction plot of interaction site of docking between 7COM (Main protease) and the following phytochemicals: A. 1,3,6 Trigalloyl glucose, B. Arjunetin, C. Arjungenin, D. Arjunic acid, E. Arjunolic acid, F. Beta-sitosterol, G. Chebulic acid, H. Daucosterol, I. Ellagic acid, J. Isoquercitrin, K. Isorhamnetin, L. Luteolin, M. Quercetin, N. Rutin and O. Terminolic acid. All the interactions represented by different colour schemes are shown at the right hand side bottom corner in the figure.

The main protease plays a key role in mediating viral transcription and replication [15]. This is considered as one of the most important enzymes of SARS CoV 2 and used as primary drug target in several studies [15]. The crystal structure of Main protease (PDB ID: 7COM) was retrieved and subjected to docking against the library of fifteen phytochemicals. All of them bound to active site of protein [15] with highly favourable ACE (<-200 kcal/mol) except Ellagic acid. Among these, 1,3,6-Trigalloyl glucose was found to be the best inhibitor with lowest ACE (−421.03 kcal/mol). It mainly interacts with hydrophobic residues Met49, Leu50, Pro168, Gln192, Met165, Asp187, His41 residing in the predicted binding pocket (Fig. 7A; Supplementary Table 12). Arjunetin (−363.46 kcal/mol), Arjungenin (−316.44 kcal/mol), Arjunolic acid (−303.57 kcal/mol), Beta-Sitosterol (−317.28 kcal/mol), Daucosterol (−350.94 kcal/mol), Terminolic acid (−346.53 kcal/mol) also exhibit significant inhibitory properties as evident from their ACE data and show favourable molecular interaction with the crystal structure of main protease. All of them mainly interact through hydrophobic interaction as observed in the 2D interaction plot (Fig. 7) and Supplementary Table 12.

### 3.9 RNA dependent RNA polymerase (RdRp) – (6M71) as drug target

**Fig. 8:**
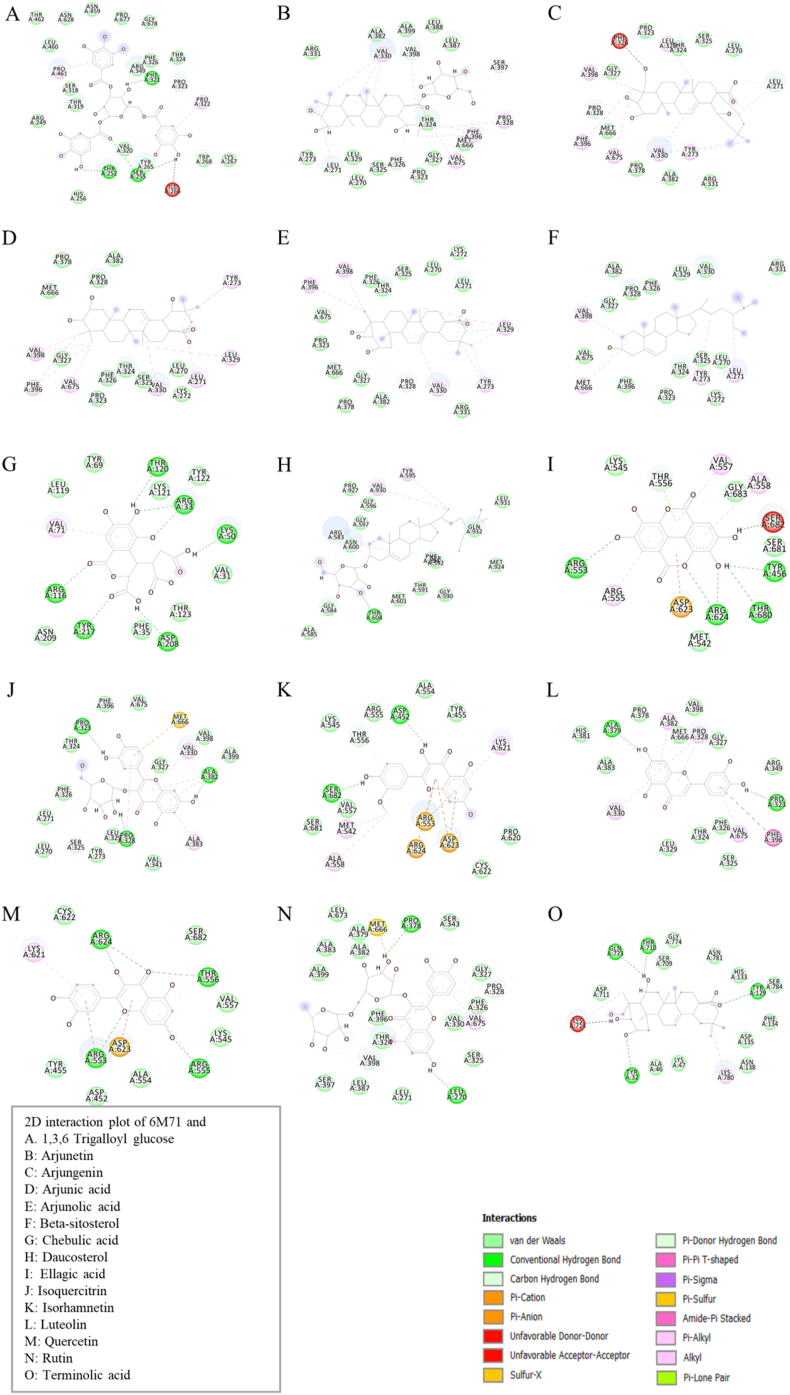
2D interaction plot of interaction site of docking between 6M71 (RNA-dependent RNA polymerase (RdRp) NSP12) and the following phytochemicals: A. 1,3,6 Trigalloyl glucose, B. Arjunetin, C. Arjungenin, D. Arjunic acid, E. Arjunolic acid, F. Beta-sitosterol, G. Chebulic acid, H. Daucosterol, I. Ellagic acid, J. Isoquercitrin, K. Isorhamnetin, L. Luteolin, M. Quercetin, N. Rutin and O. Terminolic acid. All the interactions represented by different colour schemes are shown at the right hand side bottom corner in the figure.

The RNA-dependent RNA polymerase (RdRp) NSP12 catalyses synthesis of viral RNA with the help of two cofactors NSP7 and NSP8, playing a crucial role in viral replication and transcription [16]. Our docking study against the crystal structure of NSP12 (PDB ID: 6M71) based on ACE data revealed that Arjunetin is the best inhibitor (−404.57 kcal/mol) against NSP12 (Supplementary Table 13). Arjunetin mainly interacts with the highly hydrophobic residues of NSP12 i.e. Val330, Val398, Leu271, Val675, Phe396, Pro328 (Fig. 8B, Supplementary Table 13). Arjungenin (−370.67 kcal/mol), Arjunic acid (−385.79 kcal/mol), Arjunolic acid (−364.58 kcal/mol), Beta-Sitosterol (−340.7 kcal/mol), Daucosterol (−303.09 kcal/mol) and Isoquercitrin (−330.31 kcal/mol) also show high inhibitory properties (Supplementary Table 13). In case of Arjungenin, Arjunic acid, Arjunolic acid and Beta-Sitosterol, the nature of interaction is completely hydrophobic as evident from the 2D interaction plot (Fig. 8C, D, 8E and 8F respectively) and Supplementary Table 13. Daucosterol and Isoquercitrin interact through both H-bonding and alkyl hydrophobic interactions (Fig. 8H and J, Supplementary Table 13). In 6M71, most of these molecules are found to bind to the interface domain of the RdRp. The interface domain serves as a connector between NiRAN and polymerase domain. This domain is also known to work as binding partner for Nsp8 protein of the poly-protein complex [25]. Binding of these molecules to this domain may hinder the formation of polyprotein complex and also disturb the connectivity between the functional domains.

### 3.10 1,3,6 trigalloyl glucose, Beta-Sitosterol and Daucosterol as multi-target drug

Overall, based on the ACE data (Supplementary Fig. 1) for all fifteen phytochemicals it is visible that ACE values of 1,3,6 Trigalloyl glucose and Beta-sitosterol are significantly lower compared to other twelve ligands, i.e. −332.14 ± 55.74 and −324.75 ± 36.98 kcal/mol respectively. Daucosterol is also highly effective inhibitor based on very low ACE data (−335.67 ± 104.79 kcal/mol) for all proteins except for NSP13 Helicase (PDB ID: 6ZSL). Apart from these, the ACE values of Arjunetin, Arjungenin, Arjunic acid and Arjunolic acid suggest that they also exhibit promising inhibitory effect against six proteins out of eight. According to ADME/T studies (Supplementary Table 5), Arjunetin and 1,3,6 Trigalloyl glucose are not suitable as conventional drugs.

## 4 Discussion

In our study, we chose the path of *blind* docking in which there would not be any flexibility in the side chain of any amino acid of the target protein and the binding pocket was not defined in order to avoid the biased approach of the ligand towards the active site. Our blind docking approach relies primarily on the ACE data and feasible intermolecular interactions between ligand and protein target. Docking results identified 1,3,6-Trigalloyl glucose, Beta-Sitosterol and Daucosterol as the multi-target inhibitory drug against SARS-CoV-2 proteins of important biological functions.

1,3,6-Trigalloyl glucose belongs to the gallotanin category. The structural analysis of all docking interactions of 1,3,6-Trigalloyl glucose with all eight protein targets revealed that the hydroxyl groups of gallic acid moieties are responsible for H-bonding and pi cloud of benzene ring involves in pi–alkyl interaction (Fig. 1, Fig. 2, Fig. 3, Fig. 4, Fig. 5, Fig. 6, Fig. 7, Fig. 8A). Beta-Sitosterol and Daucosterol belong to plant sterol category. Beta-Sitosterol possesses highly hydrophobic steroid moiety with hydroxyl group at 3-position and Daucosterol is the glucoside of Beta-sitosterol at 3-position. Beta-Sitosterol interacts mainly through its four hydrophobic fused aliphatic rings. Similar to Beta-Sitosterol, Daucosterol also interacts through its hydrophobic steroid rings (Fig. 1, Fig. 2, Fig. 3, Fig. 4, Fig. 5, Fig. 6, Fig. 7, Fig. 8F). Due to this reason, the interactions in these two cases are highly hydrophobic in nature. In addition to that, Daucosterol involves in H-bonding through its hydroxyl group of glucose ring (Fig. 1, Fig. 2, Fig. 3, Fig. 4, Fig. 5, Fig. 6, Fig. 7, Fig. 8H). Arjunetin, Arjungenin, Arjunic acid and Arjunolic acid belong to Triterpenoid category. Structural analysis of docking poses of these compounds indicates that these molecules interact through its six fused aliphatic hexagonal carbon rings which are completely hydrophobic in nature (Fig. 1, Fig. 2, Fig. 3, Fig. 4, Fig. 5, Fig. 6, Fig. 7, Fig. 8 B-E respectively). These compounds are also involved in H-bonding through their exposed hydroxyl groups and carboxylic groups. In case of Arjunetin, it possesses glycosyl group in addition to Triterpenoid moiety, which actively involves in H-bonding (Fig. 1, Fig. 2, Fig. 3, Fig. 4, Fig. 5, Fig. 6, Fig. 7, Fig. 8B). It was observed that the docking interactions of these above-mentioned ligands are primarily hydrophobic in nature which suggests that these ligands readily access the hydrophobic groove of the target proteins through their hydrophobic groups. Because of these highly favourable hydrophobic interactions, the ACE becomes highly negative which confirms the irreversible spontaneous nature of interaction.

In general, galloyl glucose molecules are known for their anti-cancer and anti-diabetic activities [26]. To the best of our knowledge, there is no report on antiviral activity of 1,3,6-Trigalloyl glucose, however, it is known to protect the bone marrow-derived mesenchymal stem cells (bmMSCs) against erastin-induced ferroptosis [27]. In this study, for the first time, 1,3,6-Trigalloyl glucose is identified as antiviral multi-target drug.

Beta-Sitosterol is known to exhibit immune-modulating, anti-inflammatory, anti-ulcer, anti-diabetic, and anti-cancer activities [[28], [29], [30]]. Beta-Sitosterol exhibits antiviral activity against fowlpox and herpes viruses [29] which suggested that it is having the potential to be the antiviral drug. Lin *el al.* reported that Beta-Sitosterol exhibits inhibitory effect (IC_50_: 1210 μM) on proteolytic activity of the SARS-CoV 3CL^pro^ as evident from the cell-based assay [31].

Daucosterol is reported to exhibit anti-cancer activities [30]. It is reported that Daucosterol acts as neuroprotective agent against Oxygen-Glucose Deprivation/Reperfusion-mediated injury and reduces somatic cell loss and apoptotic rate [32]. Although there is no clear report on antiviral activity of Daucosterol, the antiviral application of daucosterol and sitosterol is patented in CN201310488412.XA [33].

Arjunetin is known to possess anti-diabetic activity through inhibition of alpha-amylase accelerator [34]. A recent report on molecular docking studies showed that arjunetin binds to the SARS-CoV-2 protease (3CL, PL and RdRP) and exhibit higher binding affinity compared to that of FDA approved protease inhibitor drugs Lopinavir and Remdesivir, which supports our claim on arjunetin as potential multi-target drug against SARS-CoV-2 proteins [35].

Arjunic acid and Arjunolic acid exhibit anti-inflammatory property by reducing Nitric Oxide production [36]. In a recent report, Arjunic Acid is identified as potential inhibitors of SARS-CoV-2 (Mpro) using docking studies of saponins and tannins [37], which supports our finding, however, in our study it is found to be a potential multi-target drug against of SARS-CoV-2 proteins. Although there is a report on inhibitory effects of Arjunolic acid from *Cochlospermum tinctorium* on Epstein–Barr virus activation [38], there is no clear conventional report on antiviral activity of this phytochemical against SARS-CoV-2 proteins. In a recent report, Sherif et al. studied the antiviral activity of 26 active polyphenolic compounds of *Rhus* spp. against SARS-CoV-2 main protease enzyme (M^pro^; 6LU7) using molecular docking approach and identified six polyphenolic compounds as potential inhibitors based on drug likeness, solubility in water, and synthetic accessibility score (SAS) analysis [39]. These polyphenols mainly interact through H-bonding, whereas, in our study, 1,3,6-Trigalloyl glucose, Beta-Sitosterol and Daucosterol interact primarily through hydrophobic residues along with H-bonding.

Instead of administering one type of drug, the combination of two or three phytochemicals can be tried in this type of treatment, which requires further wet-lab experimental validation. Side effects of these components also have to be analysed *in vivo*, although there is no notable side effects of *T. chebula* reported yet except diarrhea in some patients when administered in excess [40]. Although extensive wet-lab experimental validation both *in vitro* and *in vivo* is required in future to make it applicable in reality, our study provides the insight into the application of phytochemicals from *T. chebula* in the treatment and prevention of COVID-19 which can help the scientific and health care communities further to develop effective drugs from plants.

## 5 Conclusion

In our study, we investigated the potential of various types of phytochemicals which can be extracted from *T. chebula* and can be used as multi-target inhibitors against many functional proteins of SARS-CoV-2. *Ayurvedic* or herbal medicine have been in practice for thousands of years and still is used in treatment of a wide variety of disorders using naturally derived products. This motivated us to explore the field of *Ayurvedic* medicines in order to find multi-target inhibitors for SARS-CoV-2 and we found *T. chebula* to be our answer, which has both antibacterial and antiviral properties.

Using computational analysis, we observed that 1,3,6-Trigalloyl glucose, Beta-Sitosterol and Daucosterol possess the most promising potential as effective inhibitors against all eight proteins of SARS-CoV-2. Apart from these; Arjunetin, Arjungenin, Arjunic acid and Arjunolic acid also exhibited promising inhibitory effect against six proteins out of the eight. Although 1,3,6-Trigalloyl glucose and Arjunetin are not having drug like properties as per the ADME/T studies, these two can be modified to make it applicable as these are having great potential as effective inhibitors against SARS-CoV-2. The use of naturally derived compounds have their own set of benefits and have a huge potency to be used as antiviral drugs, and hence more research should be done in this domain.

## Author contributions

**Arkaniva Sarkar:** Conceptualization, Methodology, Software, Visualization, Investigation, Project administration, Writing - Original draft preparation. **Rushali Agarwal:** Software, Data curation, Visualization, Investigation. **Boudhayan Bandyopadhyay:** Conceptualization, Methodology, Supervision, Investigation, Writing- Reviewing and Editing.

## Source of funding

None.

## Conflict of interest

None.
