# Supplementary material for: Molecular docking studies of phytochemicals from Terminalia chebula for identification of potential multi-target inhibitors of SARS-CoV-2 proteins
Source: J Ayurveda Integr Med. 2022 Feb 16;13(2):100557. doi: 10.1016/j.jaim.2022.100557 (PMC8847108; doi:10.1016/j.jaim.2022.100557)
Supplement: Multimedia component 1 [file mmc1.docx]

**Supplementary Information**

**Molecular docking studies of phytochemicals from *Terminalia chebula* for identification of potential multi-target inhibitors of SARS-CoV-2 proteins**

Arkaniva Sarkar^1^, Rushali Agarwal^1^, Boudhayan Bandyopadhyay^2*^

^1^ School of Bioscience, Engineering and Technology, VIT Bhopal University, Madhya Pradesh, India

^2^Department of Biotechnology, School of Life Science and Biotechnology, Adamas University, Kolkata, India

**TABLES**

Supplementary Table 1. List of phytochemicals from *T. chebula* used in this study.

| **Ligand** | **Chemical Formula** | **Chemical structure** | **Molecular weight** | **Category** |
| --- | --- | --- | --- | --- |
| 1,3,6-Trigalloyl glucose | C_27_H_24_O_18_ | 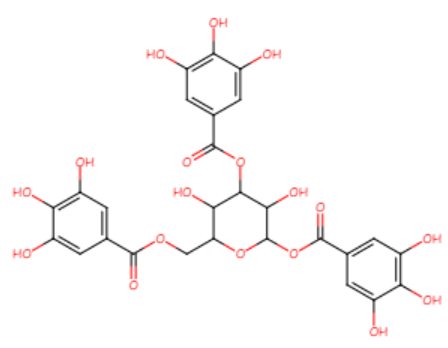 | 636.47 | Gallotanin |
| Arjunetin | C_36_H_58_O_10_ | 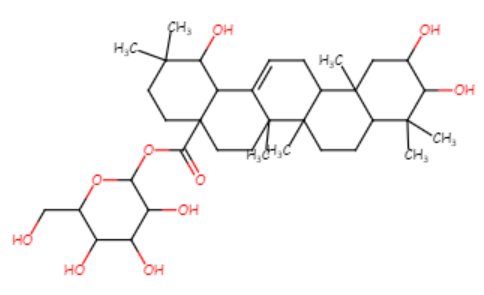 | 650.84 | Triterpenoid |
| Arjungenin | C_30_H_48_O_6_ | 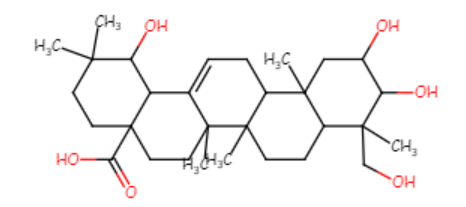 | 504.7 | Triterpenoid |
| Arjunic acid | C_30_H_48_O_5_ | 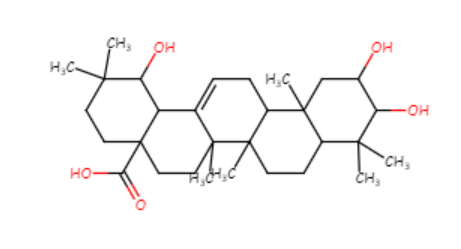 | 488.7 | Triterpenoid |
| Arjunolic acid | C_30_H_48_O_5_ | 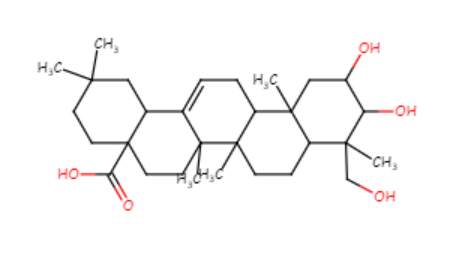 | 488.7 | Triterpenoid |
| Beta-Sitosterol | C_29_H_50_O | 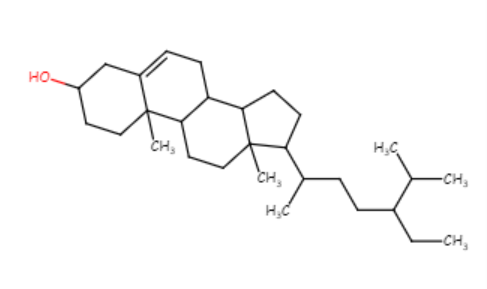 | 414.71 | Phytosterol |
| Chebulic acid | C_14_H_12_O_11_ | 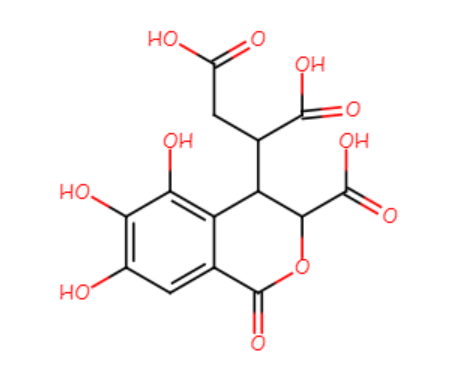 | 356.24 | Ellagitannin |
| Daucosterol | C_35_H_60_O_6_ | 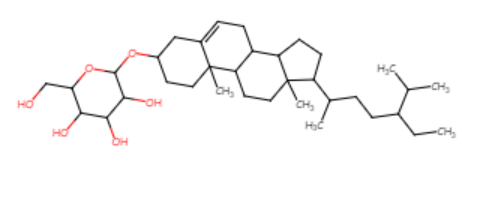 | 576.85 | Phytosterol |
| Ellagic acid | C_14_H_6_O_8_ | 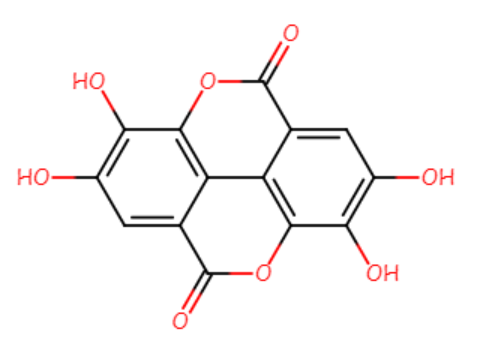 | 302.19 | Tannin |
| Isoquercitrin | C_21_H_20_O_12_ | 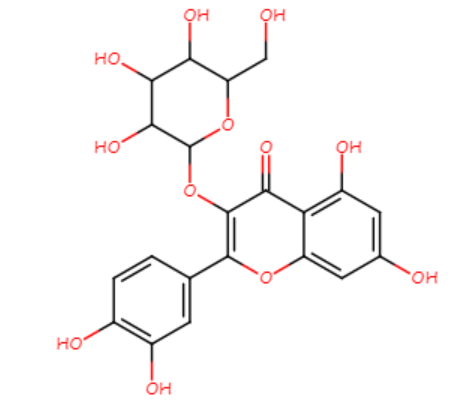 | 464.38 | Flavonoid |
| Isorhamnetin | C_16_H_12_O_7_ | 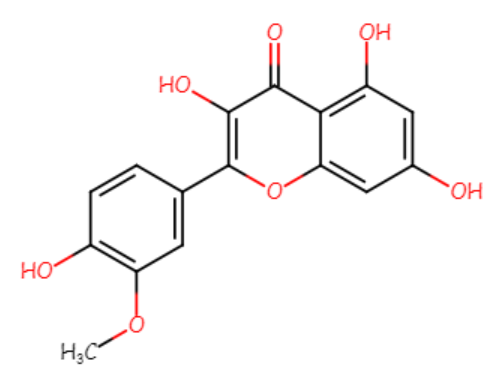 | 316.26 | Flavonoid |
| Luteolin | C_15_H_10_O_6_ | 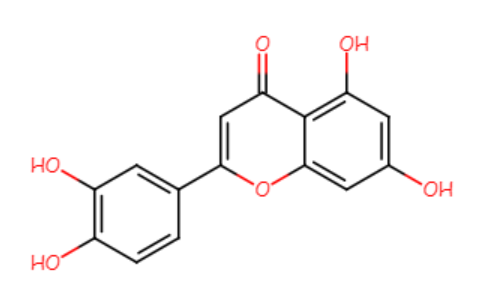 | 286.24 | Flavonoid |
| Quercetin | C_15_H_10_O_7_ | 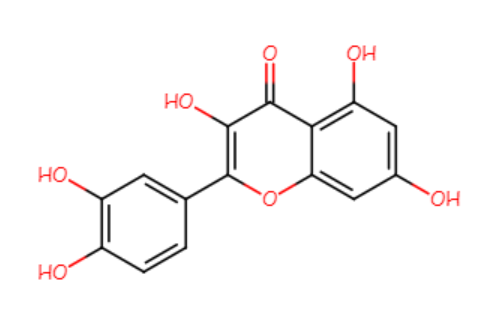 | 302.24 | Flavonoid |
| Rutin | C_27_H_30_O_16_ | 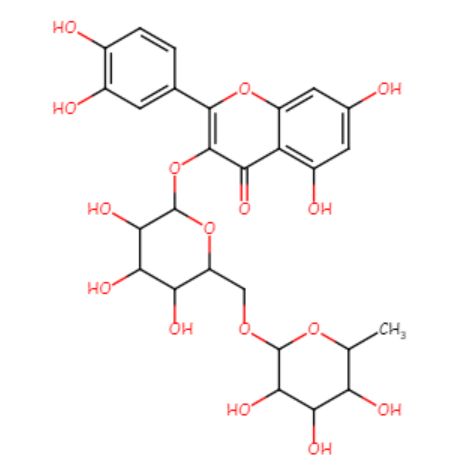 | 610.52 | Flavonoid |
| Terminolic acid | C_30_H_48_O_6_ | 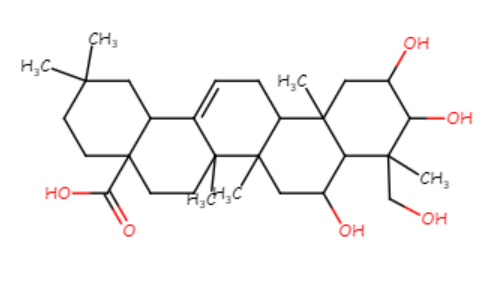 | 504.7 | Triterpenoid |

Supplementary Table 2. The list of the target proteins of SARS-CoV-2 along with their PDB ID, chain ID and chain length

| **Proteins** | **PDB id** | **Chains** | **Chain length** |
| --- | --- | --- | --- |
| Nucleocapsid protein N-terminal RNA binding domain | 6M3M | A,B,C,D | 136 |
| NSP15 Endoribonuclease | 6VWW | A,B | 370 |
| Nsp9 RNA binding protein | 6W4B | A,B | 117 |
| Papain-like protease | 6WUU | A,B,C,D | 326 |
| Nonstructural protein 10 (nsp10) | 6ZCT | A | 125 |
| Helicase | 6ZSL | A,B | 603 |
| Main protease | 7COM | A,B | 306 |
| SARS-Cov-2 RNA-dependent RNA polymerase | 6M71 | A | 942 |

Supplementary Table 3. Evaluation of the quality of PDB files of the target proteins of SARS-CoV-2

| **PDB ID** | Z score | % residues in favoured | % residues in outliers |
| --- | --- | --- | --- |
| 6M3M | -5.06 | 96.48 | 0.00 |
| 6VWW | -7.94 | 97.83 | 0.00 |
| 6W4B | -4.25 | 94.12 | 0.00 |
| 6WUU | -8.77 | 95.34 | 0.08 |
| 6ZCT | -3.73 | 94.31 | 0.00 |
| 6ZSL | -8.52 | 96.15 | 0.17 |
| 7COM | -7.36 | 98.15 | 0.00 |
| 6M71 | -12.25 | 95.77 | 0.00 |

Supplementary Table 4: Binding pocket prediction for the crystal structures of target proteins of SARS-CoV-2

| **Protein** | **Pocket no.** | **Volume** | **Surface** | **Enclosure** | **Hydrophobicity ratio** | **Drug score** |
| --- | --- | --- | --- | --- | --- | --- |
| 6M3M | P1 | 277.12 | 365.39 | 0.26 | 0.35 | 0.59 |
|  | P0 | 312.9 | 707.83 | 0.22 | 0.32 | 0.57 |
|  | P2 | 235.33 | 354.95 | 0.27 | 0.28 | 0.48 |
| 6VWW | P0 | 680.02 | 756.69 | 0.08 | 0.25 | 0.85 |
|  | P3 | 263.92 | 297.8 | 0.11 | 0.34 | 0.74 |
|  | P1 | 411.21 | 666.35 | 0.14 | 0.56 | 0.68 |
| 6W4B | P1 | 564.35 | 818.37 | 0.16 | 0.42 | 0.78 |
|  | P0 | 577.92 | 1014.28 | 0.22 | 0.55 | 0.77 |
| 6WUU | P1 | 677.38 | 879.85 | 0.09 | 0.25 | 0.85 |
|  | P0 | 783.1 | 1023.19 | 0.14 | 0.45 | 0.83 |
|  | P3 | 283.71 | 386.14 | 0 | 0.12 | 0.68 |
| 6ZCT | P0 | 392.06 | 409.3 | 0.18 | 0.45 | 0.64 |
|  | P1 | 176.9 | 284.4 | 0.11 | 0.33 | 0.41 |
|  | P4 | 106.56 | 319.45 | 0.16 | 0.81 | 0.25 |
| 6ZSL | P1 | 684.82 | 827.54 | 0.12 | 0.36 | 0.85 |
|  | P2 | 613.86 | 801.63 | 0.19 | 0.46 | 0.81 |
|  | P0 | 686.74 | 754.76 | 0.11 | 0.29 | 0.8 |
| 7COM | P0 | 1043.78 | 1408.64 | 0.15 | 0.52 | 0.81 |
|  | P1 | 574.91 | 697.21 | 0.19 | 0.41 | 0.74 |
|  | P2 | 297.6 | 558.95 | 0.26 | 0.53 | 0.58 |
| 6M71 | P2 | 513.04 | 633.32 | 0 | 0.09 | 0.88 |
|  | P1 | 526.05 | 651.08 | 0.02 | 0.18 | 0.86 |
|  | P6 | 406.14 | 664.96 | 0.14 | 0.22 | 0.81 |

Supplementary Table 5. ADME/T Properties of the phytochemicals used in this study

Supplementary Table 5.1. Pharmacokinetic properties

| **Compound Name** | **Lipinski Rule of Five** | | **TPSA^(^**[**^Bhowmik et al., 2020^**](#_ENREF_3)**^)^** | **AB%^(^**[**^Bhowmik et al., 2020^**](#_ENREF_3)**^)^** | **Solubility** |
| --- | --- | --- | --- | --- | --- |
|  | **Properties** | **Values** |  |  |  |
| 1,3,6-Trigalloyl glucose | Molecular weight(g/mol) | 636.47 | 310.66 | 1.82 | Soluble |
|  | Lipophilicity | 0.36 |  |  |  |
|  | Hydrogen Bond Donors | 11 |  |  |  |
|  | Hydrogen Bond Acceptors | 18 |  |  |  |
|  | Molar Refractivity | 142.86 |  |  |  |
|  | Lipinski’s Violations | 3 |  |  |  |
| Arjunetin | Molecular weight(g/mol) | 650.84 | 177.14 | 47.88 | Moderately Soluble |
|  | Lipophilicity | 3.36 |  |  |  |
|  | Hydrogen Bond Donors | 7 |  |  |  |
|  | Hydrogen Bond Acceptors | 10 |  |  |  |
|  | Molar Refractivity | 170.95 |  |  |  |
|  | Lipinski’s Violations | 2 |  |  |  |
| Arjungenin | Molecular weight(g/mol) | 504.7 | 118.22 | 68.21 | Moderately Soluble |
|  | Lipophilicity | 4.5 |  |  |  |
|  | Hydrogen Bond Donors | 5 |  |  |  |
|  | Hydrogen Bond Acceptors | 6 |  |  |  |
|  | Molar Refractivity | 140.14 |  |  |  |
|  | Lipinski’s Violations | 1 |  |  |  |
| Arjunic Acid | Molecular weight(g/mol) | 488.7 | 97.99 | 75.19 | Poorly Soluble |
|  | Lipophilicity | 5.17 |  |  |  |
|  | Hydrogen Bond Donors | 4 |  |  |  |
|  | Hydrogen Bond Acceptors | 5 |  |  |  |
|  | Molar Refractivity | 138.98 |  |  |  |
|  | Lipinski’s Violations | 0 |  |  |  |
| Arjunolic Acid | Molecular weight(g/mol) | 488.7 | 97.99 | 75.19 | Poorly Soluble |
|  | Lipophilicity | 5.84 |  |  |  |
|  | Hydrogen Bond Donors | 4 |  |  |  |
|  | Hydrogen Bond Acceptors | 5 |  |  |  |
|  | Molar Refractivity | 138.98 |  |  |  |
|  | Lipinski’s Violations | 0 |  |  |  |
| Beta-Sitosterol | Molecular weight(g/mol) | 414.71 | 20.23 | 102.02 | Poorly Soluble |
|  | Lipophilicity | 9.34 |  |  |  |
|  | Hydrogen Bond Donors | 1 |  |  |  |
|  | Hydrogen Bond Acceptors | 1 |  |  |  |
|  | Molar Refractivity | 133.23 |  |  |  |
|  | Lipinski’s Violations | 1 |  |  |  |
| Chebulic Acid | Molecular weight(g/mol) | 356.24 | 198.89 | 40.38 | Very Soluble |
|  | Lipophilicity | -0.81 |  |  |  |
|  | Hydrogen Bond Donors | 6 |  |  |  |
|  | Hydrogen Bond Acceptors | 11 |  |  |  |
|  | Molar Refractivity | 75.99 |  |  |  |
|  | Lipinski’s Violations | 2 |  |  |  |
| Daucosterol | Molecular weight(g/mol) | 576.85 | 99.38 | 74.71 | Poorly Soluble |
|  | Lipophilicity | 7.74 |  |  |  |
|  | Hydrogen Bond Donors | 4 |  |  |  |
|  | Hydrogen Bond Acceptors | 6 |  |  |  |
|  | Molar Refractivity | 165.61 |  |  |  |
|  | Lipinski’s Violations | 1 |  |  |  |
| Ellagic Acid | Molecular weight(g/mol) | 302.19 | 141.34 | 60.23 | Soluble |
|  | Lipophilicity | 1.1 |  |  |  |
|  | Hydrogen Bond Donors | 4 |  |  |  |
|  | Hydrogen Bond Acceptors | 8 |  |  |  |
|  | Molar Refractivity | 75.31 |  |  |  |
|  | Lipinski’s Violations | 0 |  |  |  |
| Isoquercitrin | Molecular weight(g/mol) | 464.38 | 210.51 | 36.37 | Soluble |
|  | Lipophilicity | 0.36 |  |  |  |
|  | Hydrogen Bond Donors | 8 |  |  |  |
|  | Hydrogen Bond Acceptors | 12 |  |  |  |
|  | Molar Refractivity | 110.16 |  |  |  |
|  | Lipinski’s Violations | 2 |  |  |  |
| Isorhamnnetin | Molecular weight(g/mol) | 316.26 | 120.36 | 67.47 | Soluble |
|  | Lipophilicity | 1.87 |  |  |  |
|  | Hydrogen Bond Donors | 4 |  |  |  |
|  | Hydrogen Bond Acceptors | 7 |  |  |  |
|  | Molar Refractivity | 82.5 |  |  |  |
|  | Lipinski’s Violations | 0 |  |  |  |
| Luteolin | Molecular weight(g/mol) | 286.24 | 111.13 | 70.66 | Soluble |
|  | Lipophilicity | 2.53 |  |  |  |
|  | Hydrogen Bond Donors | 4 |  |  |  |
|  | Hydrogen Bond Acceptors | 6 |  |  |  |
|  | Molar Refractivity | 76.01 |  |  |  |
|  | Lipinski’s Violations | 0 |  |  |  |
| Quercetin | Molecular weight(g/mol) | 302.24 | 131.36 | 63.68 | Soluble |
|  | Lipophilicity | 1.54 |  |  |  |
|  | Hydrogen Bond Donors | 5 |  |  |  |
|  | Hydrogen Bond Acceptors | 7 |  |  |  |
|  | Molar Refractivity | 78.03 |  |  |  |
|  | Lipinski’s Violations | 0 |  |  |  |
| Rutin | Molecular weight(g/mol) | 610.52 | 269.43 | 16.04 | Soluble |
|  | Lipophilicity | -0.33 |  |  |  |
|  | Hydrogen Bond Donors | 10 |  |  |  |
|  | Hydrogen Bond Acceptors | 16 |  |  |  |
|  | Molar Refractivity | 141.38 |  |  |  |
|  | Lipinski’s Violations | 3 |  |  |  |
| Terminolic Acid | Molecular weight(g/mol) | 504.7 | 118.22 | 68.21 | Moderately Soluble |
|  | Lipophilicity | 4.5 |  |  |  |
|  | Hydrogen Bond Donors | 5 |  |  |  |
|  | Hydrogen Bond Acceptors | 6 |  |  |  |
|  | Molar Refractivity | 140.14 |  |  |  |
|  | Lipinski’s Violations | 1 |  |  |  |

Supplementary Table 5.2. Pharmacodynamic profile

| **Ligands** | **Blood brain barrier (BBB)** | **Human intestinal absorbtion (HIA)** | **Caco2 permeability** | **P-glycoprotein substrate \| inhibitor** | **CYP450 3A4 Substrate \| inhibitor** | **Ames toxicity** | **Carcinogenicity** | **Acute oral toxicity** | **Rat acute toxicity LD50, mol/kg** | **hERG** |
| --- | --- | --- | --- | --- | --- | --- | --- | --- | --- | --- |
| 1,3,6-Trigalloyl glucose | BBB+ | HIA- | Caco2- | Substrate/ Non-inhibitor | Non substrate/ Non-inhibitor | Non AMES toxic | Non- carcinogens | III | 2.6316 | Weak inhibitor |
| Arjunetin | BBB+ | HIA+ | Caco2- | Substrate/ Non-inhibitor | Substrate/ Non-inhibitor | Non AMES toxic | Non- carcinogens | III | 2.8073 | Weak inhibitor |
| Arjungenin | BBB+ | HIA+ | Caco2- | Substrate/ Non-inhibitor | Non substrate/ Non-inhibitor | Non AMES toxic | Non- carcinogens | III | 2.1598 | Weak inhibitor |
| Arjunic acid | BBB+ | HIA+ | Caco2+ | Substrate/ Non-inhibitor | Substrate/ Non-inhibitor | Non AMES toxic | Non- carcinogens | III | 2.1021 | Weak inhibitor |
| Arjunolic acid | BBB+ | HIA+ | Caco2- | Substrate/ Non-inhibitor | Substrate/ Non-inhibitor | Non AMES toxic | Non- carcinogens | III | 2.1598 | Weak inhibitor |
| Beta-Sitosterol | BBB+ | HIA+ | Caco2+ | Substrate/ Inhibitor | Substrate/ Non-inhibitor | Non AMES toxic | Non- carcinogens | I | 2.6561 | Weak inhibitor |
| Chebulic acid | BBB- | HIA+ | Caco2- | Substrate/ Non-inhibitor | Non substrate/ Non-inhibitor | Non AMES toxic | Non- carcinogens | IV | 2.6663 | Weak inhibitor |
| Daucosterol | BBB+ | HIA+ | Caco2- | Substrate/ Inhibitor | Substrate/ Non-inhibitor | Non AMES toxic | Non- carcinogens | III | 2.9113 | Weak inhibitor |
| Ellagic acid | BBB+ | HIA+ | Caco2- | Substrate/ Non-inhibitor | Non substrate/ Non-inhibitor | Non AMES toxic | Non- carcinogens | II | 2.6213 | Weak inhibitor |
| Isoquercitrin | BBB- | HIA+ | Caco2- | Substrate/ Non-inhibitor | Non substrate/ Non-inhibitor | AMES toxic | Non- carcinogens | III | 2.3869 | Weak inhibitor |
| Isorhamnetin | BBB- | HIA+ | Caco2+ | Substrate/ Non-inhibitor | Non substrate/ inhibitor | Non AMES toxic | Non- carcinogens | III | 2.7192 | Weak inhibitor |
| Luteolin | BBB- | HIA+ | Caco2- | Substrate/ Non-inhibitor | Non substrate/ inhibitor | Non AMES toxic | Non- carcinogens | II | 3.02 | Weak inhibitor |
| Quercetin | BBB- | HIA+ | Caco2- | Substrate/ Non-inhibitor | Non substrate/ inhibitor | Non AMES toxic | Non- carcinogens | II | 3.02 | Weak inhibitor |
| Rutin | BBB- | HIA+ | Caco2- | Substrate/ Non-inhibitor | Non substrate/ Non-inhibitor | Non AMES toxic | Non- carcinogens | III | 2.4984 | Weak inhibitor |
| Terminolic acid | BBB+ | HIA+ | Caco2- | Substrate/ Non-inhibitor | Substrate/ Non-inhibitor | Non AMES toxic | Non- carcinogens | III | 2.1598 | Weak inhibitor |

Supplementary Table 6: Docking results of 6M3M (N-terminal RNA binding domain of Nucleocapsid protein)

| **Ligand** | **Atomic contact energy**  **(kcal/mol)** | **Residues involved in H-bond** | **Residues involved in other interactions** |
| --- | --- | --- | --- |
| 1,3,6-Trigalloyl glucose | -325.98 | GLY138, ALA139, ASN141 | PRO68, ARG69, ALA135, ILE85, ALA139 |
| Arjunetin | -115.01 | TRP133, TYR124, ALA135, ARG69, PRO68, LYS66 |  |
| Arjungenin | -273.95 |  | ALA174 |
| Arjunic acid | -150.56 | LYS128, TRP133, ARG69 | TYR124, LYS66 |
| Arjunolic acid | -217.25 | PRO68 | ILE132, TRP133, ARG69, TYR124 |
| Beta-Sitosterol | -326.98 |  | LEU168, LEU162, VAL159 |
| Chebulic acid | -25.99 | ASP64, ASN127, TRP133 |  |
| Daucosterol | -358.36 |  | ILE75 |
| Ellagic acid | -252.11 | ASN141 | PRO123, LEU114, ALA120, PRO143 |
| Isoquercitrin | -106.67 | LYS66, ASP129 | ASP64, ILE132, TRP133 |
| Isorhamnetin | -133.39 | ARG69 | TRP133, ALA135, LYS66 |
| Luteolin | -232.77 | TYR124, ASN141 | LEU114, PRO143, ALA120 |
| Quercetin | -58.67 | ASP64, ASP129, GLY130 | TRP133, ASN127 |
| Rutin | -270.47 | LEU168, PRO163, LEU162, LEU160 | ALA174, LEU168 |
| Terminolic acid | -90.89 | ASN127, TYR124, TRP133 | TRP133 |

Supplementary Table 7: Docking results of 6VWW (NSP15 Endoribonuclease)

| **Ligand** | **Atomic contact energy (kcal/mol)** | **Residues involved in H-bond** | **Residues involved in other interactions** |
| --- | --- | --- | --- |
| 1,3,6-Trigalloyl glucose | -339.28 | SER98, PRO94, PRO271, THR48, THR49 | ILE97, ALA93, ARG91, LEU50 |
| Arjunetin | -294.42 | ASP297, TYR279 | PHE259, PHE264, LEU201, LEU266, LYS90 |
| Arjungenin | -297.74 | GLY157, VAL156, PRO154 | PHE195, VAL156 |
| Arjunic acid | -281.34 | THR193 | PHE195, VAL156 |
| Arjunolic acid | -327.48 | ASN46 | ALA93, LEU50, PRO94, ILE97 |
| Beta-Sitosterol | -343.98 |  | ILE80, ILE97, LEU50, ALA93 |
| Chebulic acid | -180.25 | THR49, ARG91, ALA95 | ALA93, ARG91, THR49 |
| Daucosterol | -332.89 | THR49, THR48 | HIS96, ILE97, PRO94, ALA93, LEU50 |
| Ellagic acid | -61.63 | SER274, THR196, ASP297 | LEU252, LYS277, TYR279 |
| Isoquercitrin | -74.73 | TYR279, SER274, ARG199, SER198 | ASN200, LYS90 |
| Isorhamnetin | -213.7 | GLN189, LEU190 | PRO191, LEU190, VAL156 |
| Luteolin | -244.27 | GLN189, THR193 | LEU190 |
| Quercetin | -43.16 | ASP297, GLU69, THR167 | LEU252, LYS277, TYR279, GLU69, LYS71 |
| Rutin | -84.56 | ASP268, LYS90 | LYS71, LYS90, VAL295, LEU252, TYR279, LYS277 |
| Terminolic acid | -73.4 |  |  |

Supplementary Table 8: Docking results of 6W4B (Non-structural protein 9: Nsp9)

| **Ligand** | **Atomic contact energy (kcal/mol)** | **Residues involved in H-bond** | **Residues involved in other interactions** |
| --- | --- | --- | --- |
| 1,3,6-Trigalloyl glucose | -286.51 | LYS93, THR36 | PHE41, ILE92, ARG40 |
| Arjunetin | -464.74 | THR35, GLY39, ASN34 | PHE41, VAL42, ARG40, LYS37 |
| Arjungenin | -301.06 | VAL42, PRO58, SER60 | PHE57, ARG40, VAL42 |
| Arjunic acid | -389.73 | THR36, ARG40 | ILE66, PHE57, ARG40, LYS37 |
| Arjunolic acid | -374.82 |  | ILE66, ARG40, LYS37 |
| Beta-Sitosterol | -256.52 |  | ARG40 , ILE66 , ILE92 , PHE41 , VAL42 , PHE57 |
| Chebulic acid | -220.89 | CYS74, LEU104 | PHE76, CYS74 |
| Daucosterol | -409.83 | ALA108, VAL111 | LEU89 , LEU104, CYS74, PHE91 , PHE76, LEU113 , ALA108 |
| Ellagic acid | -241.95 | LEU104, ARG75 | LEU113, LEU104, LEU107, CYS74 |
| Isoquercitrin | -268.47 | ARG40, PRO58 | ARG40 |
| Isorhamnetin | -205.55 | SER60 | VAL42, ILE66, PHE41, MET13 , PHE57 |
| Luteolin | -181.15 | PRO58 | ARG40, ILE66 , VAL42, PHE57 |
| Quercetin | -253.8 |  | LEU89 , CYS74 , ALA108 , LEU104 |
| Rutin | -291.09 | PRO58, THR36 | PHE57, ARG40, GLY39 |
| Terminolic acid | -229.54 |  | MET13, ARG40 , PHE41, ILE92, ILE66 |

Supplementary Table 9: Docking results of 6WUU (Papain-like protease)

| **Ligand** | **Atomic contact energy (kcal/mol)** | **Residues involved in H-bond** | **Residues involved in other interactions** |
| --- | --- | --- | --- |
| 1,3,6-Trigalloyl glucose | -260.13 | GLU214, LYS217, TYR233, ILE314, THR311 | THR313, THR312, ASN186, VAL188, TYR233, LYS217 |
| Arjunetin | -38.97 | THR54, GLY52, TYR71, TYR56 | LEU16, LEU87 |
| Arjungenin | -82.2 | ALA131, TYR71 | TYR71, LEU87 , LYS91 |
| Arjunic acid | -106.41 | THR54, ASP37, ASN13, TYR71, ARG138 | TYR71, TYR56, ALA135 |
| Arjunolic acid | -42.1 | TYR71, ALA131 | TYR71, LEU87, LYS91 |
| Beta-Sitosterol | -363.05 | THR311 | ILE314, VAL188 , TYR233 |
| Chebulic acid | 3.97 | ASN13, TYR56, TYR83, ASN146, TYR72, TYR71 |  |
| Daucosterol | -520.75 | CYS111 | CYS111, LEU162, CYS270, TYR264, TYR268 |
| Ellagic acid | -1.49 | ARG138 | ALA135 , GLU143, TYR56, ASN146 |
| Isoquercitrin | -69.35 | ASN13, ALA135, ALA131, ASN146, TYR71, ASP12, ARG138 | ALA135, ALA131, ASN146, TYR56, TYR71 |
| Isorhamnetin | -255.64 | ASN267, HIS272, CYS111 | CYS270, LEU162, CYS111 |
| Luteolin | -116.77 | ASP37, TYR71 | TYR56, TYR72 |
| Quercetin | -256.38 | THR225 | ILE222 , VAL187, CYS189 , CYS192 , CYS224 |
| Rutin | -26.14 | GLY38, ASP37, TYR56, TYR71, ALA135 | GLU143, LEU87, ASN146 |
| Terminolic acid | -56.25 | TYR56, TYR72, TYR83 | LYS91, ALA135 |

Supplementary Table 10: Docking study of 6ZCT (Non-structural protein 10: nsp10)

| **Ligand** | **Atomic contact energy (kcal/mol)** | **Residues involved in H-bond** | **Residues involved in other interactions** |
| --- | --- | --- | --- |
| 1,3,6-Trigalloyl glucose | -397.95 | PRO86, PRO84, CYS90, CYS74 | LYS87, ALA24, PRO84, CYS74, PRO86 |
| Arjunetin | -302.42 | ILE55 | CYS74, TYR76, HIS83, LEU92, VAL116 |
| Arjungenin | -467.1 | CYS90, PRO84 | LEU92, CYS74, HIS83, TYR76, ILE81, PRO84 |
| Arjunic acid | -334.02 |  | PRO84, TYR76, LEU92, ILE55, TRP123, VAL116 |
| Arjunolic acid | -457.06 |  | LYS87, PRO86, PRO84, TYR76 , LEU112, CYS74 , LEU92, CYS90  HIS83 |
| Beta-Sitosterol | -287.57 | CYS74 | LEU92, TRP123, VAL116, CYS117 |
| Chebulic acid | -239.14 | ASN114, THR111, CYS74, CYS90, PHE89, ASP91 | ASP91 |
| Daucosterol | -227.14 | THR7 | LYS113, LEU31, PRO37, CYS103, PHE110, TYR126 |
| Ellagic acid | -185.36 | THR111 | LEU92 |
| Isoquercitrin | -344.28 | ASP82, ALA23 | PHE19, ALA18, ILE81, ALA20 |
| Isorhamnetin | -149.1 | CYS74, THR111 | LEU92 , PRO86 |
| Luteolin | -208.12 | CYS74, VAL116 | ILE55, CYS74, LEU92, VAL116 |
| Quercetin | -245.96 | LEU112, CYS73 | LEU92, CYS74 , TYR76, LEU112 |
| Rutin | -359.1 | TYR76, THR111, CYS90 | PHE89, VAL116, LEU92, ILE55, CYS90, ASP91 |
| Terminolic acid | -456.93 | LYS87 | PRO84, TRP123, LEU92, VAL116 |

Supplementary Table 11: Docking study of 6ZSL (Helicase)

| **Ligand** | **Atomic contact energy (kcal/mol)** | **Residues involved in H-bond** | **Residues involved in other interactions** |
| --- | --- | --- | --- |
| 1,3,6-Trigalloyl glucose | -339.91 | ALA208, ASP483 | LYS202 , ALA520, ILE488, PRO175, VAL484, LEU176 |
| Arjunetin | -393.3 | SER229, ALA140, VAL232 | HIS230, MET233, VAL232 |
| Arjungenin | -314.33 | ALA208, ASP207 | VAL484, PRO175 |
| Arjunic acid | -316.8 | ARG173 | PRO175, VAL484, LEU176, LYS202, VAL209 |
| Arjunolic acid | -379.29 | LEU165 | LEU176, PRO175, VAL484, LLYS202 |
| Beta-Sitosterol | -361.93 |  | TRP167, LEU176, VAL484, PRO175 |
| Chebulic acid | -12.91 | GLN404, ARG567, ARG443, LYS288, GLY285 | ASP374, ALA316, ALA313 |
| Daucosterol | -182.33 | GLU375, ALA313, ALA312 | ALA316, ARG442, ALA313 |
| Ellagic acid | -226.72 | LYS202 | VAL484, PRO175, ASP483, LYS202 , LEU176 |
| Isoquercitrin | -49.29 | GLY287 | ALA316, ARG443 |
| Isorhamnetin | -16.58 | ASP374, GLU375 | ARG443, LYS288, ALA313, ALA316 |
| Luteolin | -245.53 | ASP483, PRO174, LEU165, ASP207 | LEU176, LEU165,VAL209, VAL484 |
| Quercetin | -223.56 | ASP207 | VAL484, VAL209, LEU176 |
| Rutin | -277.51 | GLY415, ARG409, LEU417, ASN557 | ARG560, PRO406, ARG409, LEU412 |
| Terminolic acid | -92.44 | GLU540, ASP374 | HIS290, LYS288, LYS320 |

Supplementary Table 12: Docking study of 7COM (Main protease)

| **Ligand** | **Atomic contact energy (kcal/mol)** | **Residues involved in H-bond** | **Residues involved in other interactions** |
| --- | --- | --- | --- |
| 1,3,6-Trigalloyl glucose | -421.03 | GLN189 | MET49, LEU50 , PRO168, GLN192, MET165, ASP187, HIS41 |
| Arjunetin | -363.46 | GLN189 | HIS41, ALA191, PRO168, MET49 |
| Arjungenin | -316.44 | HIS164, GLN189 | HIS41, MET49 , MET165, PRO168 |
| Arjunic acid | -244.41 | ASN142, SER144 | HIS41, TYR118, LEU141, CYS145 |
| Arjunolic acid | -303.57 | ARG188, CYS145 | MET49, MET165, HIS172, HIS163 |
| Beta-Sitosterol | -317.28 | THR26 | HIS41 , PRO168 , MET165, CYS145, LEU27 |
| Chebulic acid | -205.97 | GLN189, GLN192 |  |
| Daucosterol | -350.94 | GLN192 | HIS41, MET49 , CYS145, MET165, PRO168, ALA191 |
| Ellagic acid | -145.12 | GLY71, LYS97 | PRO122, ALA70 |
| Isoquercitrin | -265.22 | SER144, CYS145, THR25 | MET49, CYS145 , MET165 , HIS41 |
| Isorhamnetin | -248.96 | TYR118, CYS145, GLU166 | LEU141, SER144, HIS163, CYS145 |
| Luteolin | -249.37 | HIS164, GLN192 | PRO169, MET165, HIS41 |
| Quercetin | -205.26 | GLU166, GLN192 | MET49, ARG188, PRO168 , MET165 |
| Rutin | -205.74 | LEU141, SER144, THR26 | CYS145, MET49 , HIS41 |
| Terminolic acid | -346.53 | HIS41, HIS164 | MET49, MET165, LEU167, PRO168 |

Supplementary Table 13: Docking study of 6M71 (RNA-dependent RNA polymerase (RdRp) NSP12)

| **Ligand** | **Atomic contact energy (kcal/mol)** | **Residues involved in H-bond** | **Residues involved in other interactions** |
| --- | --- | --- | --- |
| 1,3,6-Trigalloyl glucose | -286.33 | PHE321, SER255, THR252 | PRO461 , PRO322, THR252 |
| Arjunetin | -404.57 |  | VAL330, VAL398, LEU271, VAL675, PHE396, PRO328 |
| Arjungenin | -370.67 |  | LEU271,TYR273, VAL330,VAL675, PHE396,VAL398, LEU329 |
| Arjunic acid | -385.79 |  | VAL398,PHE396, VAL675,VAL330, LEU271,LEU329, TYR273 |
| Arjunolic acid | -364.58 |  | PHE396, VAL398, LEU329 , TYR273, VAL330 |
| Beta-Sitosterol | -340.70 |  | VAL398 , MET666 , TYR273, LEU271 |
| Chebulic acid | -73.32 | THR120, ARG33, LYS50, ASP208, TYR217, ARG116 | VAL71 |
| Daucosterol | -303.09 | THR604 | TYR595 , VAL930 , ARG583 |
| Ellagic acid | -50.98 | ARG553, ARG624, THR680, TYR456 | ARG555, ASP623, ALA558, VAL557, THR556 |
| Isoquercitrin | -330.31 | PRO323, ALA382, PRO328 | MET666 , VAL330 , ALA382 , ALA383 , PRO328 |
| Isorhamnetin | 46.43 | SER682, ASP452 | THR556, LYS621, MET542, ALA558 , ARG553, ARG624, ASP623 |
| Luteolin | -264.39 | ALA379, PRO323 | PHE396, ALA379, ALA382, PRO328, VAL330, VAL675 |
| Quercetin | 67.47 | ARG624, THR556, ARG555, ARG553 | LYS621, THR556, ARG553, ASP623 |
| Rutin | -382 | PRO378, LEU270 | MET666, VAL675 , VAL398 |
| Terminolic acid | -60.69 | GLN773, THR710, TYR129, TYR32 | LYS714, LYS780 |

**FIGURE**

**
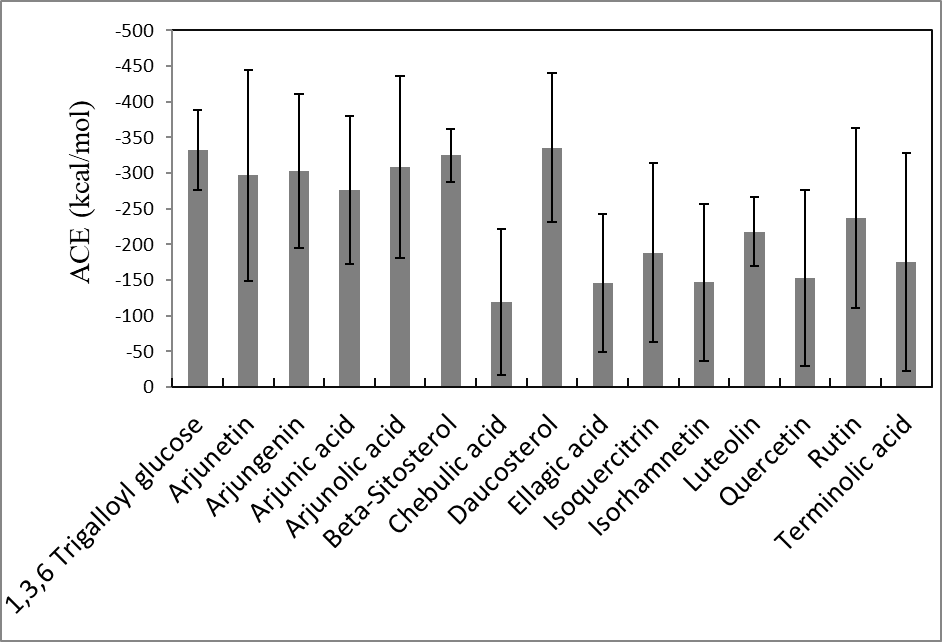
**

Supplementary Fig 1. Average value of Atomic Contact Energy (ACE) of docking interactions of ligands. Error bar represents the standard deviation of average ACE values of individual phytochemical from docking analysis conducted against all eight proteins.
